# PMF proteins mediate mitochondrial fusion in Arabidopsis

**DOI:** 10.1073/pnas.2601242123

**Published:** 2026-04-22

**Authors:** Ryan P. Kenneally, Yu Tang, Wesley J. Bobst, Rui Tong Khor, Lily Garibyan, Neha R. Jag, Yangnan Gu

**Affiliations:** ^a^Department of Plant and Microbial Biology, University of California, Berkeley, CA 94720

**Keywords:** mitochondrial fusion, outer mitochondrial membrane protein, Promoter of Mitochondria Fusion 1/2, liquid–liquid phase separation, megamitochondria

## Abstract

Mitochondrial dynamics—continuous fission and fusion—are hallmarks of these organelles and essential for mitochondrial function across eukaryotes. While fission is relatively well understood in plants, the mechanism of fusion remains incompletely defined. In animals and fungi, dynamin-related proteins are emerging fusion factors, but their plant homologs do not localize to mitochondria, suggesting plants rely on a distinct fusion mechanism. Here, we identify PMF1 (Promoter of Mitochondrial Fusion 1) and its paralog PMF2 as plant-specific outer mitochondrial membrane proteins that promote mitochondrial fusion. PMF abundance directly correlates with fusion capacity: overexpression markedly enlarges mitochondria and increases fusion rates, producing megamitochondria, whereas *pmf1 pmf2* mutants show reduced mitochondrial volume and severe fusion defects under hypoxic stress. We further show that PMF1’s cytoplasmic, intrinsically disordered N terminus undergoes liquid–liquid phase separation, a property necessary and sufficient to promote mitochondria fusion in vivo. These results reveal PMF-mediated protein condensation as a kingdom-specific mechanism regulating mitochondrial dynamics in plant cells.

## Significance

Although mitochondrial fusion is critical for sustaining mitochondrial function, the molecular mechanism that drives this process is divergent across eukaryotes and remains poorly defined in plants. In this study, we identify a previously uncharacterized family of plant mitochondrial outer membrane proteins that promote fusion through an amphipathic helix and phase separation of their intrinsically disordered domains. This work reveals a molecular and biophysical strategy for regulating mitochondrial dynamics in plant cells.

Mitochondria are double membrane-bound organelles descended from α-proteobacteria and are ubiquitous in eukaryotes. They house their own genome (mtDNA) and play a pivotal role in cellular bioenergetics through oxidative phosphorylation, generating the majority of cellular ATP. However, mitochondrial functions extend far beyond energy production; they are central to diverse metabolic pathways, including lipid biosynthesis (1, 2), iron-sulfur and heme cluster production (3), and calcium homeostasis and signaling (4). In plants, mitochondria also metabolize glycine, contributing to photorespiration byproduct reutilization (5). Moreover, they function as key signaling hubs, orchestrating redox balance (6), reactive oxygen species (ROS) signaling (7), innate immunity (7, 8), and programmed cell death (9).

Mitochondrial dynamics, characterized by frequent fission and fusion events, are a hallmark of these organelles and facilitate mtDNA and protein exchange (10, 11). This property has been suggested to play an important role in plants, whose mitochondrial genomes are often composed of multiple, nonstoichiometric subgenomes, and individual mitochondria often contain incomplete subgenome sets (12, 13). Fission-fusion equilibrium also profoundly influences mitochondrial morphology, resulting in diverse structures ranging from discrete puncta to elongated linear forms, branching networks, and large globular bodies. Notably, plant mitochondria typically appear as discrete puncta, whereas animal mitochondria more often exhibit elongated, tubular, or networked morphologies. This distinction reflects a unique balance between fusion and fission processes in plant cells, in which fission predominates over fusion. Plant mitochondrial morphology is known to adapt to cellular conditions, exhibiting increased fusion during germination (14) and metabolic stress (15, 16), and increased fission prior to cytokinesis to ensure equitable inheritance (17). Mitochondrial morphology influences metabolic capacity in plant and mammalian cells, with fragmented mitochondria displaying reduced membrane potential and ATP production (18, 19), as well as dysregulated ROS production (19–21). Dysregulation of mitochondrial dynamics in humans has been implicated in diverse pathologies, including lung adenocarcinoma and breast cancer (22), chronic kidney disease (23), metabolic disorders like type 2 diabetes (24), and neurodegenerative conditions, including Alzheimer’s, Parkinson’s, and Huntington’s diseases (25).

Mechanisms underlying mitochondrial fusion have been reported in multiple model systems (26, 27). For instance, fusion of the outer mitochondrial membrane (OMM) is mediated by dynamin-related GTPases Mitofusin 1/2 (MFN1/2) in humans and Fzo1p in yeasts (28, 29). MFN1/2 monomers bind GTP, causing a conformational change that stabilizes trans-dimerization between proximal OMMs; subsequent GTP hydrolysis results in an additional conformational change that brings membranes together and destabilizes them, inducing OMM fusion (30, 31). MFN1/2 also contain lipid-bilayer-binding amphipathic helices in their Heptad Repeat 1 domain, and disruption of these helices greatly diminishes MFN’s ability to facilitate mitochondrial fusion (32–34). Intriguingly, the plant homolog of these GTPases, Fzo-like (FZL), has been reported to localize to the inner chloroplast membrane and function in modulating thylakoid organization, rather than mitochondrial fusion (35, 36). Although frequent fusion events have been observed in plant cells, no plant dynamin-related proteins have been directly linked to mitochondrial fusion, suggesting the existence of an alternative, plant-specific fusion mechanism.

Three proteins have been implicated in regulating plant mitochondrial fusion. The first protein, FRIENDLY, a member of the CLUSTERED MITOCHONDRIA (CLU) superfamily, modulates intermitochondrial association. Disruption of FRIENDLY results in clusters of punctate mitochondria with prolonged association (37, 38). While fusion rates appear elevated in *friendly* mutants, clustered mitochondria in *friendly* plants show no evidence of membrane or matrix continuity, suggesting that FRIENDLY may regulate mitochondrial interactions, either prefusion association or postfission separation, rather than the fusion process itself. The precise role of FRIENDLY in the fusion–fission cycle remains to be determined. *At*Miro1/2 are OMM Ras-superfamily GTPases and represent strong candidates for the functional counterparts of human MFN1/2. Miro2 overexpression results in fewer but larger mitochondria and increases mitochondrial association with the endoplasmic reticulum (ER), and its GTPase activity is necessary to promote mitochondrial fusion (39). Knockout of *Miro2*’s homolog, *Miro1*, results in enlarged mitochondria in pollen tubes, leading to the hypothesis that Miro1 may regulate fission rather than fusion (40). However, more recent work demonstrates that Miro1 facilitates mitochondrial fusion in guard cells in response to immune activation (19). Miro1 is enriched in mitochondrial fusion sites, where it prolongs intermitochondrial contact duration (19). Miro1 oligomerization is required for its fusion-promoting activity (19). These data support a model in which Miro1/2 act as the functional counterparts of MFN1/2 in Arabidopsis and highlight the divergence of mitochondrial dynamics regulation in plants. The specific mechanisms, molecular details, and interacting partners of Miro1/2 in plants remain largely unknown.

In this study, we characterized two mitochondrial transmembrane proteins in *Arabidopsis thaliana* that share sequence homology and are exclusively found in land plants. These proteins, designated Promoter of Mitochondrial Fusion 1 (PMF1) and PMF2, localize specifically to the OMM where they function as positive regulators of mitochondrial fusion. We demonstrated that PMF protein expression levels correlate directly with the extent of mitochondrial fusion. Overexpression of PMF1 dramatically increases mitochondrial size and rate of fusion, inducing anomalously large fusion-derived megamitochondria structures. In contrast, *pmf1 pmf2* double knockouts exhibit reduced mitochondrial volumes and impaired mitochondria fusion under hypoxic stress conditions, leading to decreased seedling survival. We established that PMF1’s N-terminal domain extends into the cytoplasm, and that this domain is both necessary and sufficient to promote mitochondria fusion in vivo. Notably, the PMF1 N-terminal domain is characterized by an amphipathic helix and extensive intrinsically disordered regions capable of mediating liquid–liquid phase separation in vitro; both features are essential for promoting megamitochondria formation in vivo. Fusion of PMF1’s N-terminal domain to a different OMM protein was sufficient to drive megamitochondria formation in vivo. These findings suggest that plant cells use a distinct mechanism to promote mitochondrial fusion compared to animals and fungi.

## Results

### Characterization of PMF1, a Conserved Transmembrane Protein in Land Plants.

**Fig. 1.**
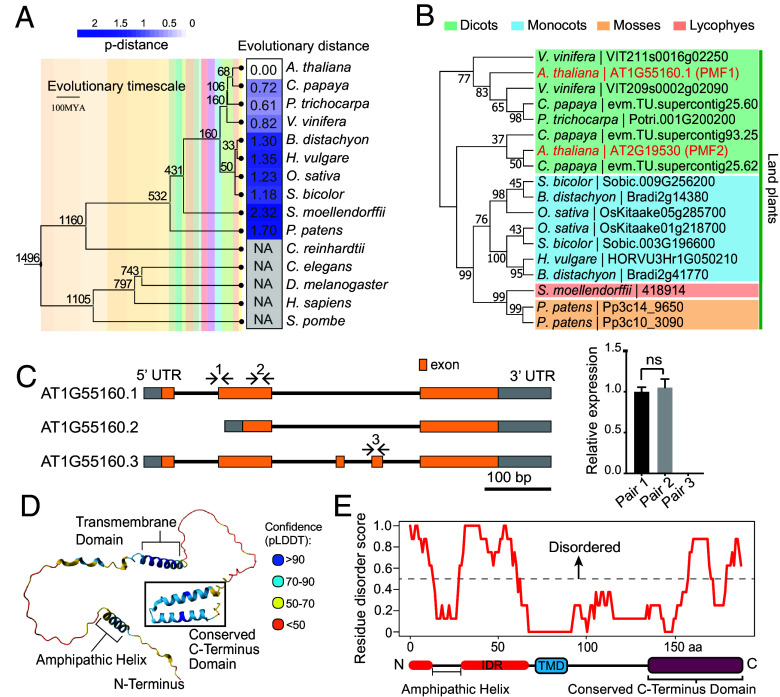
PMF1 is a land plant-specific transmembrane protein. (*A*) Heatmap of evolutionary distances of Arabidopsis *PMF1* gene and its homologs in 14 eukaryotic species. NA indicates that no homologs are identified in the species using a BLAST search. The evolutionary timescale of plant life is shown on the *Left*. Bar = 100 million years. (*B*) Phylogenetic analysis of PMF1 and its homologs in ten land plant species using protein sequences. The dendrogram and bootstrap values on each branch were generated by MEGA-X using the maximum likelihood method with 100 bootstraps. (*C*) Predicted gene splicing models for *PMF1*, with primer pair locations indicated on the models by numbers. The *Right* panel plots the relative transcript expression as quantified by the corresponding qPCR primer pairs. Data are presented as mean ± SD (n = 3 technical replicates). Statistics were performed using Student’s *t* tests. NS stands for not significant. Similar results were obtained twice. (*D*) Arabidopsis PMF1 protein structure predicted by AlphaFold3 and modeled in PyMOL3.0, with coloring based on pLDDT value predicted by AlphaFold. (*E*) Schematic representation of PMF1 protein domain architecture alongside a plot of disorder scores generated by MolPhase. A dashed line marks the disorder threshold at 0.5.

The PMF1 protein (AT1G55160) was originally identified through proximity labeling proteomics using a nuclear envelope (NE) protein as bait. This suggests that PMF1 may be an NE-associated protein or localizes to membrane contact sites between the NE and other organelles. Phylogenetic and genomic analyses revealed *PMF1* as a conserved, plant-specific gene prevalent in land plants but absent in algae and nonplant eukaryotes (Fig. 1 *A* and *B*). The evolutionary distances between *AtPMF1* and its homologs correlate with the overall plant evolutionary timeline, suggesting lineage-specific evolution and functional specialization of PMF1 and its homologs in land plants.

The *AtPMF1* gene exhibits ubiquitous expression across diverse tissues (*SI Appendix,* Fig. S1*A*) and has three predicted gene models (Fig. 1*C*). *PMF1.1* encodes a 188 amino acid (aa) protein with a putative transmembrane domain (TMD1) located near the middle of the protein. *PMF1.2* encodes an N-terminal truncated variant of *PMF1.1*, while *PMF1.3* possesses two additional small exons that result in a second predicted TMD (TMD2) adjacent to TMD1 (Fig. 1*C* and *SI Appendix,* Fig. S1*B*). Proximity labeling proteomics detected endogenous PMF1 peptides that cover the N-terminal region absent in PMF1.2, supporting the existence of PMF1.1 and/or PMF1.3 (*SI Appendix,* Fig. S1*B*). However, no peptides were detected along the putative TMDs, preventing us from determining whether PMF1.1 or PMF1.3 is the dominant isoform.

To further investigate the expression of potential *PMF1* isoforms, we designed three pairs of primers that target distinct regions of the *PMF1* gene (Fig. 1*C*) and performed quantitative RT-PCR experiments using cDNA prepared from whole seedlings of wild type (WT) Arabidopsis. We found that *PMF1.1* constitutes the predominant form of *PMF1* transcripts, with the other two potential isoforms being scarcely detectable, if present at all (Fig. 1 *C*, *Right* panel). Additionally, we did not find evidence for the expression of *PMF1.3*’s additional exons in the 75 expressed sequence tag (EST) clones available in The Arabidopsis Information Resource (TAIR) database. Collectively, these results suggest that *PMF1.1* is the predominant, if not the only, isoform of *PMF1* expressed in Arabidopsis seedlings. Henceforth, we will refer to *PMF1.1* as *PMF1*.

The majority of analyzed land plant species have two to three *PMF1* homologs, which can be roughly clustered into two distinct clades in each species (Fig. 1*B*), suggesting potential functional divergence. Arabidopsis possesses a single *PMF1* homolog (AT2G19530, e-value = 1e−18), which we denoted as *PMF2*. PMF1 and PMF2 are characterized by two conserved regions—a TMD and a 51 aa, highly conserved C-terminal region (*SI Appendix,* Fig. S1*C*), predicted by AlphaFold to form two antiparallel α-helices (Fig. 1*D*). PMF1 is predicted to be highly disordered, with approximately 76% of its residues classified as disordered by AlphaFold, indicated by predicted local distance difference test (pLDDT) scores below 50 (Fig. 1*D*) (41). Further, 75% of residues in the PMF1 N terminus are predicted by MolPhase to have disorder probability scores greater than 0.5, including a 32 aa predicted intrinsically disordered region (IDR) (Fig. 1*E*) (42). Immediately upstream of the PMF1’s N-terminal IDR, AlphaFold predicted a 16 aa α-helix. Heliquest indicates that a full-turn α-helix in this region is likely amphipathic (Fig. 1 *D* and *E* and *SI Appendix,* Fig. S1*D*). Similarly, 76% of PMF2 residues are classified as disordered by AlphaFold (*SI Appendix,* Fig. S1*E*), and the entirety of the PMF2 N terminus is predicted to be disordered by MolPhase (*SI Appendix,* Fig. S1*F*).

### PMFs Localize to the Mitochondria.

**Fig. 2.**
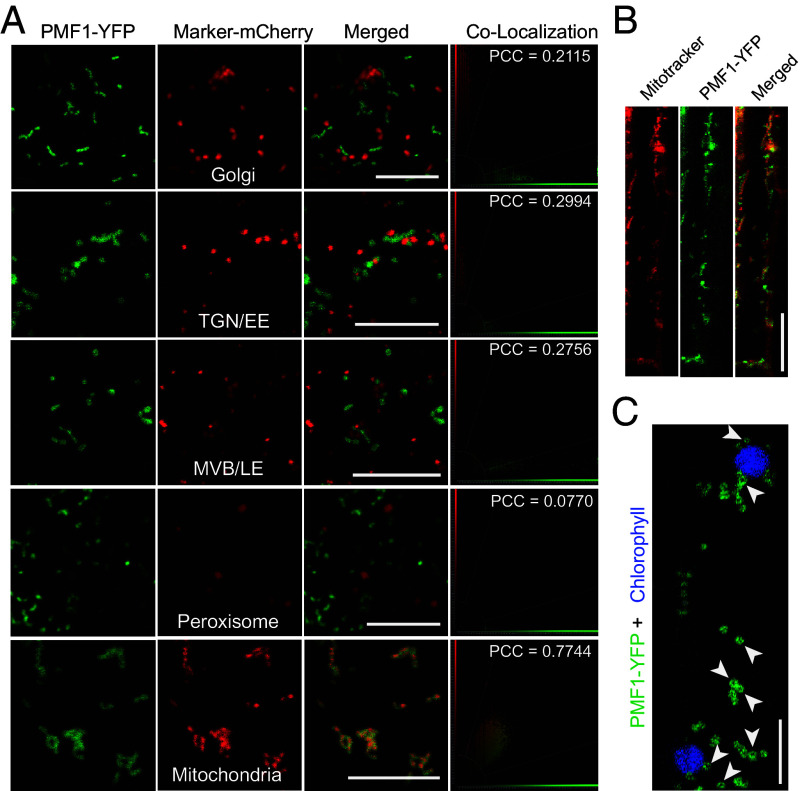
PMF1 localizes to the mitochondrial outer membrane. (*A*) Transient coexpression of PMF1-YFP and mCherry-labeled organelle markers in *Nicotiana benthamiana*. Golgi apparatus are labeled with GmMan49-mCherry, TGN/EEs are labeled with mCherry-SYP61, MVBs are labeled with mCherry-SYP21, peroxisomes are labeled with mCherry fused with peroxisomal targeting signal 1 (PST1, Ser-Lys-Leu), and mitochondria are labeled with the first 29 aa of yeast cytochrome c oxidase IV fused with mCherry. Pearson correlation coefficient (PCC) of YFP and mCherry was calculated using Imaris. (Scale bar, 10 μm.) (*B* and *C*) PMF1 localization in *35S::PMF1-YFP* transgenic Arabidopsis seedling. Hypocotyl epidermal cells were imaged. Mitochondria were stained with MitoTracker Red (*B*, Scale bar, 25 μm), and chloroplasts are pseudocolored in blue (*C*, Scale bar, 5 μm). Arrowheads indicate donut-shaped fluorescence.

To explore the in vivo localization of PMF1 protein, we fused yellow fluorescence protein (YFP) to the C-terminus of PMF1 and expressed the construct transiently under the 35S promoter in *Nicotiana benthamiana*. PMF1-YFP was found predominantly in mobile dot and rod-like structures at the cell periphery (Fig. 2*A*), indicative of endomembrane organelles. An N terminus YFP fusion (YFP-PMF1) showed a comparable localization pattern (*SI Appendix,* Fig. S2*A*). To pinpoint the specific organelle to which PMF1 is localized, we transiently coexpressed PMF1-YFP with five mCherry-tagged markers labeling different vesicular endomembrane organelles, including the Golgi, trans-Golgi network/early endosome (TGN/EE), multivesicular body (MVB), peroxisome, and mitochondria in *N. benthamiana*. We found that only the mitochondrial marker colocalizes with PMF1-YFP with a Pearson correlation coefficient (PCC) of 0.7744 (Fig. 2*A*). In line with this observation, PMF1 was previously identified as a putative mitochondrial protein based on its protein density gradient distribution (43), although PMF1 does not have a predicted Mitochondrial Targeting Signal (MTS).

The mitochondrial localization of PMF1 was further validated in *35S::PMF1-YFP* transgenic Arabidopsis seedlings stained with MitoTracker, a fluorescent probe that contains a thiol-reactive chloromethyl moiety for mitochondrial labeling (Fig. 2*B*). Notably, PMF1-YFP fluorescence exhibits a distinct donut-like pattern (Fig. 2*C* and *SI Appendix,* Fig. S2*B*), previously documented for OMM proteins, and stands in contrast to the filled fluorescent puncta characteristic of inner mitochondrial membrane (IMM) and matrix-localized proteins (1), suggesting that PMF1 is an OMM protein. This is also supported by a previous study profiling the OMM proteome with mass spectrometry, which detected PMF1 peptides in multiple replicates (1). We confirmed mitochondrial targeting of PMF1 under near-native conditions by observing colocalization of PMF1 with MitoTracker in stable *pPMF1::PMF1-GFP* transgenic lines (*SI Appendix,* Fig. S2*C*), although PMF1-GFP expression driven by the native promoter is typically weak. These findings collectively demonstrate that PMF1 localizes to mitochondria in plants and is likely to be an OMM protein.

PMF2 colocalizes with PMF1 when transiently coexpressed in *N. benthamiana* (*SI Appendix,* Fig. S2*D*). In stable transgenic lines, PMF2-YFP also colocalizes with MitoTracker-stained organelles (*SI Appendix,* Fig. S2*E*), indicating that it is also a mitochondria-localized protein.

### PMF Overexpression Leads to Formation of Megamitochondria.

**Fig. 3.**
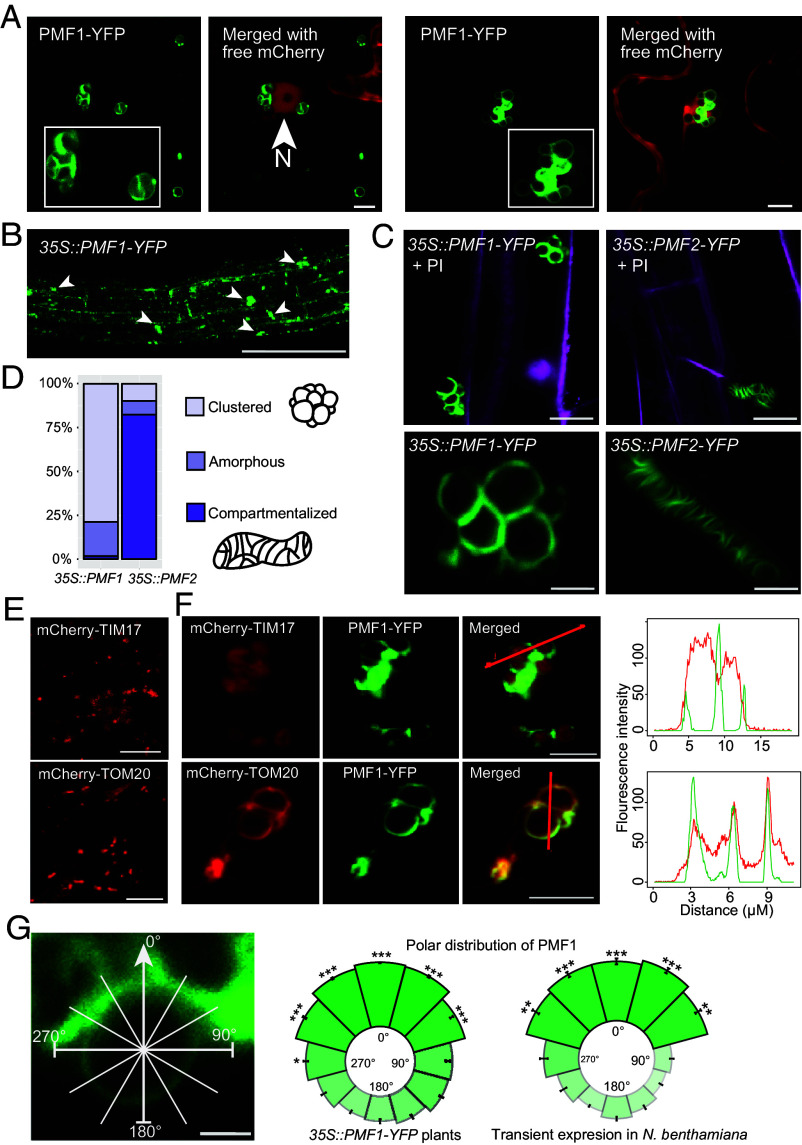
PMF1 overexpression profoundly influences mitochondrial morphology. (*A*) Transient coexpression of PMF1-YFP and free mCherry in *N. benthamiana*. The nucleus is marked by a white arrowhead. (Scale bar, 5 μm.) (*B*) Root cells in *35S::PMF1-YFP* transgenic plants. Abnormally large membrane structures are marked by white arrow heads. (Scale bar, 200 μm.) (*C*) PMF1-YFP and PMF2-YFP expressed in transgenic Arabidopsis seedlings stained with propidium iodide (PI), which is pseudocolored magenta. *Upper* and *Lower* Scale bar, 10 μm and 5 μm, respectively. (*D*) The proportion of “clustered,” “amorphous,” and “compartmentalized” morphotypes of the abnormal mitochondrial structures observed in *35S::PMF1-YFP* (n = 127) and *35S::PMF2-YFP* (n = 113) transgenic Arabidopsis seedlings. *P < 0.05 using a chi-squared test. (*E*) The IMM marker mCherry-TIM17 and the OMM marker mCherry-TOM20 localize to punctate structures when expressed alone in *N. benthamiana*. (Scale bar, 10 μm.) (*F*) PMF1-YFP coexpressed with mCherry-TIM17 or mCherry-TOM20 in *N. benthamiana*. Fluorescence intensity along the red lines was quantified and plotted on the *Right*. [Scale bar, 10 μm (*Top*) and 5 μm (*Bottom*).] (*G*) Polar distributions of PMF1-YFP on large mitochondrial structures in the *35S::PMF1-YFP* transgenic line (n = 14) and in *N. benthamiana* (n = 8). The image on the *Left* depicts how the center of the contact surface between adjacent compartments was aligned with 0° and maximum fluorescence intensity was quantified at 30° increments. *P < 0.05 compared to intensity at 180° using a Mann–Whitney *U* test. (Scale bar, 2 μm.)

In addition to PMF1’s localization to mitochondrial puncta at the cell periphery, we observed PMF1-YFP populating large circular membrane structures when overexpressed in *N. benthamiana* (Fig. 3*A*). These structures were orders of magnitude larger than typical plant mitochondria and often clustered in the perinuclear region. These structures are morphologically distinct from known plant organelles, likely representing artificial membrane structures.

We further validated the formation of these anomalous structures in transgenic Arabidopsis plants. In addition to normal punctate mitochondria, we observed clusters of circular membranes in *35S::PMF1-YFP* plants and cohesive membrane architecture compartmentalized into smaller sections in *35S::PMF2-YFP* plants (Fig. 3 *B*–*D*). These PMF-induced structures exhibited diverse morphologies, typically 5 to 10 μm in diameter, and were observed across various cell types including root and hypocotyl epidermal cells and leaf guard cells (*SI Appendix,* Fig. S3 *A* and *B*).

To validate the membrane origin of PMF-induced large membrane structures, we coexpressed PMF1-YFP with mCherry-tagged markers of both mitochondrial membranes: TOM20 for OMM and TIM17 for IMM. While these membrane markers localized exclusively to punctate mitochondria when expressed alone (Fig. 3*E*), they colocalized with the PMF1-derived large membrane structures upon coexpression (Fig. 3*F*). TOM20 exhibited near-perfect colocalization with PMF1 at the structure surface (PCC = 0.871), whereas TIM17 was observed within PMF1-YFP-populated membranes (Fig. 3*F*). This result indicates that PMF1-induced membrane structures are mitochondrial in origin, bounded by the OMM and encasing the IMM. The morphology of these large membrane structures share similarity to megamitochondria that were previously reported in multiple model organisms (29, 44, 45).

Notably, PMF1-YFP often displays markedly intensified fluorescence at the contact points between adjacent membrane-bound compartments (Fig. 3 *A* and *G*). Plant mitofusins Miro1/2 displays a similar enrichment at OMM–OMM contact sites (19, 39). This observation leads us to hypothesize that PMF1 may promote mitochondrial membrane tethering and/or subsequent fusion events, and that these megamitochondria structures arise from excessive mitochondrial fusion.

### PMF Proteins Promote Mitochondrial Fusion.

**Fig. 4.**
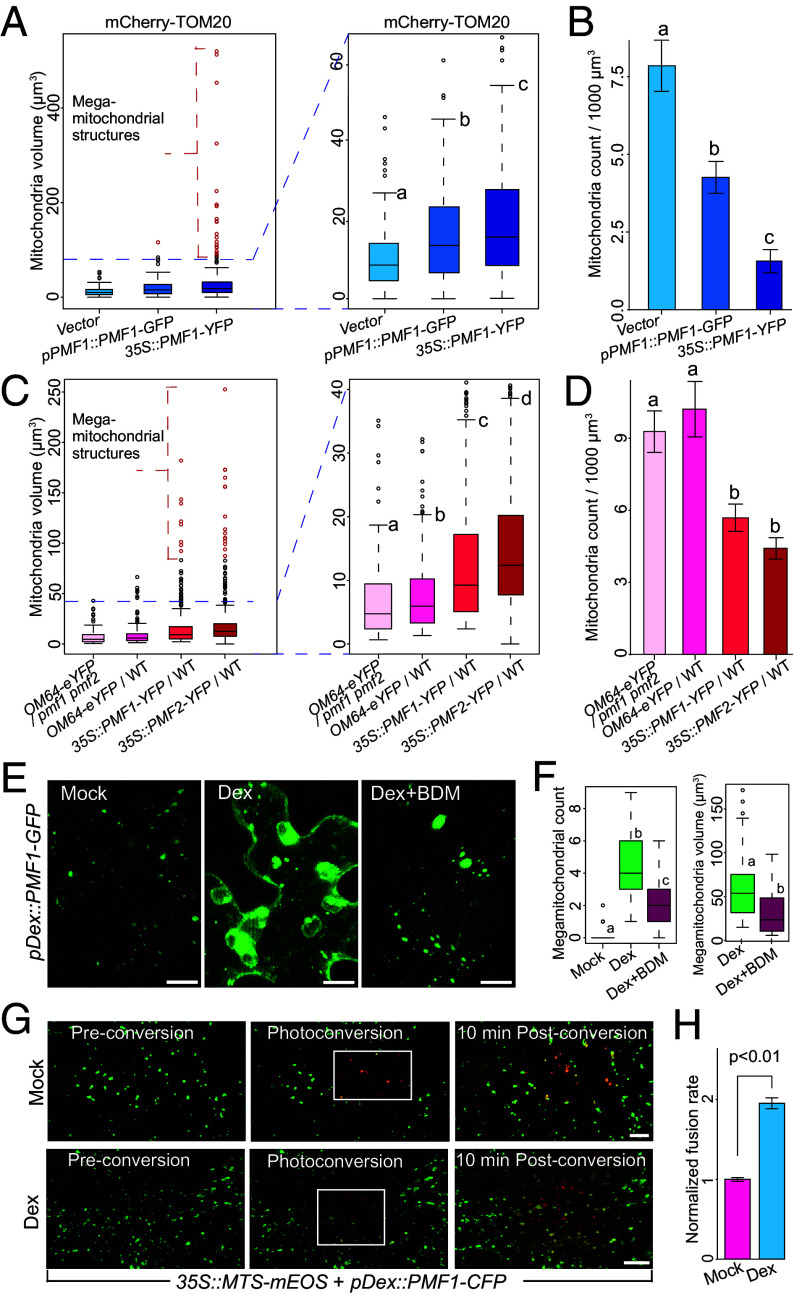
PMF1 and PMF2 promote mitochondrial fusion. (*A*) Boxplots of mitochondrial volume in *N. benthamiana* plants coexpressing *35S::mCherry-TOM20* with *pPMF1::PMF1-GFP*, *35S::PMF1-YFP,* or empty vector. Megamitochondria with volumes over 80 μm^3^ are colored in red. Statistical significance was determined using a heteroscedastic, two-tailed *t* test and a *P*-value threshold of 0.05. n > 100 for all samples. (*B*) Mitochondrial counts per 1,000 µm^3^ in *N. benthamiana* plants coexpressing *35S::mCherry-TOM20* with *pPMF1::PMF1-GFP* (n = 6), *35S::PMF1-YFP* (n = 6), or empty vector (n = 3). Statistical significance was determined using a Mann–Whitney *U* test and a *P*-value threshold of 0.05. (*C*) Boxplots of mitochondrial volume in *35S::PMF1-YFP*, *35S::PMF2-YFP*, and *35S::OM64-eYFP* transgenic Arabidopsis plants. Isogenic *35S::OM64-eYFP* lines in the *pmf1 pmf2* and WT backgrounds were used. Megamitochondria with volumes over 80 μm^3^ are colored in red. Statistical significance was determined using a heteroscedastic, two-tailed *t* test and a *P*-value threshold of 0.05. n > 150 for all samples. (*D*) Mitochondrial counts per 1,000 µm^3^ in *35S::PMF1-YFP* (n = 7), *35S::PMF2-YFP* (n = 6), and *35S::OM64-eYFP* transgenic Arabidopsis plants. Isogenic *35S::OM64-eYFP* lines in the *pmf1 pmf2* (n = 7) and WT (n = 4) backgrounds were used. Statistical significance was determined using a Mann–Whitney *U* test and a *P*-value threshold of 0.05. (*E*) Z-stack projections of *N. benthamiana* leaf epidermal cells expressing *pDex::PMF1-GFP* treated with water, dexamethasone, or dexamethasone and BDM. (Scale bar, 10 µm.) (*F*) Boxplots of megamitochondria counts (n = 75) and mitochondrial volumes in *N. benthamiana* leaf epidermal cells expressing *pDex::PMF1-GFP* treated with water, dexamethasone, or dexamethasone and BDM. For megamitochondria volume measurements, n = 36 for dexamethasone treatment and n = 34 for dexamethasone and BDM treatment. Statistical significance was determined using a heteroscedastic, two-tailed *t* test and a *P*-value threshold of 0.05. (*G*) Z-stack projections of *N. benthamiana* leaf epidermal cells coexpressing *pDex::PMF1-CFP* and *35S::MTS-mEOS* treated with water or dexamethasone. Photoconversion was induced within the boxed regions. Mitochondria exhibiting both green and red fluorescence were quantified at 0 and 10 min after photoconversion. (Scale bar, 10 µm.) (*H*) Barchart of fusion rates in mock-treated (n = 6) and dexamethasone-treated (n = 4) cells. Fusion rate was calculated as the increase in puncta displaying both green and red fluorescence 10 min after photoconversion compared to immediately after photoconversion, relative to the number of photoconverted mitochondria. Statistical significance was determined using a Mann–Whitney *U* test.

To assess PMFs’ role in mitochondrial fusion, we quantified mitochondrial count and volume using Z-stack images of *N. benthamiana* leaf epidermal cells expressing *pPMF1::PMF1-GFP* or *35S::PMF1-YFP* constructs, corresponding to weak and strong expression (*SI Appendix,* Fig. S4*A*). *mCherry-TOM20* was coexpressed to mark mitochondria and served as a control. We observed that mitochondrial volume increased by 34.4% in *pPMF1::PMF1-GFP* samples (median = 13.60 μm^3^) and 56.9% in *35S::PMF1-YFP* samples (median = 15.87 μm^3^) compared to samples expressing *mCherry-TOM20* alone (median = 10.12 μm^3^) (Fig. 4*A*). Even if we exclude the megamitochondria structures with a volume larger than 80 μm^3^, differences in mitochondrial size remained statistically significant (*P* < 0.01) between mCherry-TOM20 and both PMF1-expressing samples. Meanwhile, we observed a statistically significant decrease in average mitochondrial count per unit area in *pPMF1::PMF1-GFP* samples (count = 4.26/1,000 μm^3^) and in *35S::PMF1-YFP* samples (count = 1.56/1,000 μm^3^) compared to samples expressing *mCherry-TOM20* alone (count = 7.84/1,000 μm^3^) (Fig. 4*B*). However, the total mitochondrial volume was not significantly different between these three samples (*SI Appendix,* Fig. S4*B*). The PMF1 dosage-dependent increase in mitochondrial volume and corresponding decrease in mitochondrial count, without a change in total mitochondrial volume, is consistent with a model in which PMF1 promotes mitochondrial fusion. In line with this hypothesis, we also observed statistically significant increases in mitochondrial sphericity with elevated PMF1 expression (*SI Appendix,* Fig. S4*C*), a morphological change characteristic of enhanced fusion and previously reported for the Arabidopsis mitofusin Miro2 (39). Notably, the rounded, globular megamitochondria induced by PMF1 are distinct from the elongated morphology observed in fission-defective mutants such as *drp3a/b* and *elm1* (46, 47), suggesting a role for PMF1 in promoting mitochondrial fusion rather than inhibiting fission.

To further elucidate the functional significance of PMFs in mitochondrial fusion, we quantified mitochondrial count and volume in stable transgenic Arabidopsis lines expressing the OMM marker OM64-eYFP in isogenic WT and *pmf1 pmf2* double mutant backgrounds, as well as in *PMF1-YFP* and *PMF2-YFP* overexpression lines. PMF1 and PMF2 overexpression induced significant mitochondrial enlargement, with median volumes increasing from 5.92 μm^3^ in WT to 9.24 μm^3^ and 12.38 μm^3^, respectively. Notably, a substantial population of megamitochondria (80 to 200 μm^3^) emerged in both PMF overexpression lines (Fig. 4*C*). These increases remained statistically significant (*P* < 1e−11) even when excluding the abnormal megamitochondria structures. Conversely, the median mitochondrial size in the *pmf1 pmf2* mutant was 4.74 μm^3^ while that in WT plants was 24.8% higher (Fig. 4*C*). Although statistically significant (*P* = 0.05), this difference is modest, likely reflecting the predominance of fission over fusion in plant mitochondrial dynamics compared to yeast and mammalian systems. Indeed, this fission-biased equilibrium manifests as numerous small, punctate mitochondria in plant cells compared to the tubular mitochondrial networks observed in animal cells (48). Meanwhile, PMF1 and PMF2 overexpression significantly reduced average mitochondrial counts to 5.69/1,000 μm^3^ and 4.42/1,000 μm^3^, respectively, compared to 10.21/1,000 μm^3^ in WT (Fig. 4*D*), although the total mitochondrial volume was not significantly different between WT and either overexpression line (*SI Appendix,* Fig. S4*D*). Mitochondrial count in the *pmf1 pmf2* line showed no significant difference from WT under standard conditions (Fig. 4*D*), consistent with the low fusion activity of mitochondria in plant cells.

Mitochondrial dynamics depend on cytoskeletal activity, specifically F-actin and myosin in plants, across multiple model organisms (37, 40, 49). Conversely, mitochondrial swelling is not dependent on cytoskeletal activity. To eliminate the possibility that PMF-induced increases in mitochondrial volume are the result of mitochondrial swelling, we expressed *pDex::PMF1-GFP* in *N. benthamiana* leaf epidermal cells treated with water (Mock), 20 μM dexamethasone, or 20 μM dexamethasone plus 20 mM 2,3‐butanedione 2‐monoxime (BDM), a myosin ATPase inhibitor (50). We found that the number of megamitochondria per cell was significantly higher in dexamethasone-treated cells (average = 4.30) compared to cells treated with water (average = 0.12) or both dexamethasone and BDM (average = 2.10) (Fig. 4 *E* and *F*). The volume of megamitochondria was also significantly larger in dexamethasone-treated cells (average = 63.25 μm^3^) compared to cells treated with both dexamethasone and BDM (average = 33.68 μm^3^) (Fig. 4*F*). These findings suggest that PMF-induced increases in mitochondrial volume require cytoskeleton-dependent mitochondrial dynamics, rather than arising from mitochondrial swelling.

To directly assess the impact of PMF1 on mitochondrial fusion, we fused a mitochondrial targeting signal to a photoconvertible fluorophore (mEOS) that converts from green to red upon exposure to ultraviolet light (51). We coexpressed *pDex::PMF1-CFP* and *35S::MTS-mEOS* in *N. benthamiana* leaf epidermal cells and treated leaves with either water (Mock) or 20 μM dexamethasone to induce PMF1 expression. Subsequently, a subset of mitochondria within each cell was photoconverted by UV, and the mitochondrial fusion rate was measured by quantifying the increase in mitochondria containing both green and red fluorescence over 10 min relative to the initial number of photoconverted mitochondria. We found that dexamethasone-treated cells exhibited a nearly two-fold increase in fusion rate compared to mock-treated cells (0.027 vs 0.014 fusion events/mitochondria/minute) (Fig. 4 *G* and *H*), demonstrating that PMF1 promotes mitochondrial fusion. These data also argue against a model in which PMF1 primarily suppresses mitochondrial fission, as reduced fission would not result in the enhanced mixing of green and red fluorophores observed.

### PMFs Are Essential for Hypoxia-Induced Mitochondrial Fusion and Influence Long-Heat Stress Tolerance.

Surprisingly, *pmf1 pmf2* double mutants did not exhibit noticeable growth and developmental defects under standard conditions. In line with this observation, knockouts of plant mitofusin Miro2 have also been previously reported to display no phenotypic abnormalities under standard conditions (52). In both mutants, mitochondria remained discrete and punctate, as in WT, likely reflecting the inherently low fusion activity in plant cells.

**Fig. 5.**
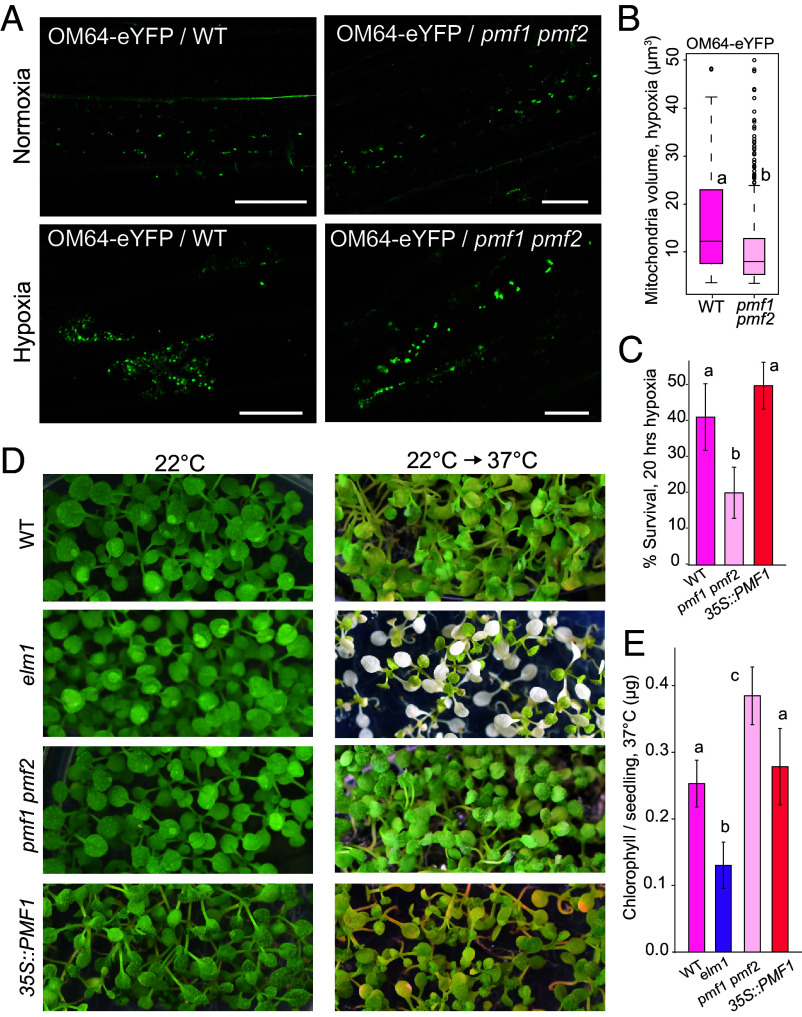
PMFs are essential for hypoxia-induced mitochondrial fusion and influence long-heat stress tolerance. (*A*) Isogenic lines of WT and *pmf1 pmf2* expressing mitochondrial marker OM64-eYFP under normoxic and hypoxic conditions. (Scale bar, 25 μm.) (*B*) Mitochondrial volumes in isogenic lines of WT and *pmf1 pmf2* expressing mitochondrial marker OM64-eYFP under hypoxic conditions. n > 100 for all samples. (*C*) Survival rate of WT, *pmf1 pmf2*, and *35S::PMF1-YFP* seedlings after 20 h of hypoxia and 3 d of recovery. Three biological replicates were analyzed for all samples, each consisting of 18 to 25 seedlings. (*D*) Seedlings of WT, *elm1*, *pmf1 pmf2*, and *35S::PMF1-YFP* grown at 22 °C (*Left*) or exposed to long-heat stress (*Right*). (*E*) Chlorophyll content per seedling of WT, *elm1*, *pmf1 pmf2*, and *35S::PMF1-YFP* plants after exposure to long-heat stress. Chlorophyll content did not differ between samples before long-heat stress. Three biological replicates were analyzed for all samples, each consisting of 16 to 40 seedlings. Statistical significance was determined using a Mann–Whitney *U* test and a *P*-value threshold of 0.05. Similar results were obtained three times.

To demonstrate the contribution of PMFs to mitochondrial fusion, we subjected WT and *pmf1 pmf2* seedlings to hypoxia, a treatment known to induce mitochondrial fusion in plants and other model organisms (15, 16, 53). While WT mitochondria exhibited the previously reported morphological shift toward semitubular sheets and clusters, we observed that *pmf1 pmf2* mutants maintained relatively normal mitochondrial morphology (Fig. 5 *A* and *B*). Both *pPMF1::PMF1-GFP* and *pPMF2::PMF2-GFP* fully complemented the mitochondrial dynamics defects observed in the *pmf1 pmf2* mutant under hypoxia (*SI Appendix,* Fig. S5*A*). Consistent with these cell-level phenotypes, 1-wk-old *pmf1 pmf2* seedlings exposed to 20 h of hypoxia followed by 3 d of recovery survived at roughly half the rate (20.11%) of WT seedlings (41.23%) (Fig. 5*C*). Together, these results underscore the functional importance of PMF1 and PMF2 in promoting mitochondrial fusion and highlight their critical role in hypoxic stress adaptation.

Mitochondrial dynamics are a well-established component of the Arabidopsis response to prolonged heat stress, typically defined as exposure to 37 °C for 3 to 5 d (47, 54). Fission-defective mutants, such as *elm1* and *drp3a/3b,* have been reported to be sensitive to such conditions (47, 54). To determine the potential role of PMF1 in long-heat stress tolerance, we subjected 10-d-old seedlings of WT, *elm1*, *pmf1 pmf2*, and *35S::PMF1-YFP* to 37 °C for 5 d followed by another 5 d of recovery. Although all genotypes grew normally under standard conditions, *elm1* seedlings exhibited pronounced chlorosis after heat stress, consistent with previous reports, whereas *pmf1 pmf2* seedlings showed less chlorosis than WT (Fig. 5 *D* and *E*). *pmf1 pmf2* mutants also had significantly larger rosette sizes after recovery compared with WT and *elm1* mutants (*SI Appendix,* Fig. S5*B*). In contrast, PMF1-overexpression lines were not significantly different from WT and did not display the rapid chlorosis characteristic of fission-defective mutants, reinforcing our conclusion that PMF1 promotes megamitochondrion formation by enhancing fusion rather than suppressing fission.

These findings suggest that mitochondrial fusion functions as a conditional adaptive mechanism in plants, becoming essential under metabolic challenges that require mitochondrial remodeling and morphological plasticity.

### PMF1 Is an OMM Protein With a Cytoplasmic N Terminus.

**Fig. 6.**
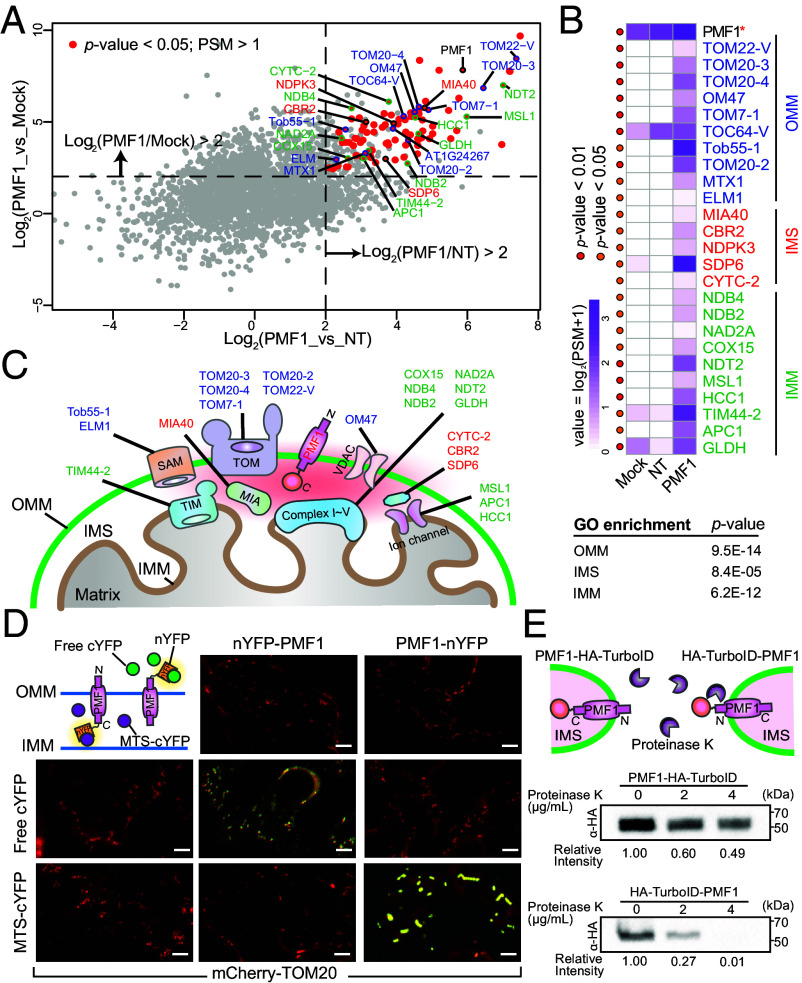
PMF1 proxiome and topology. (*A*) Proximity labeling proteomics using PMF1 as bait. Transgenic plants expressing PMF1 with C-terminus translational fusion of BioID2 were sampled. Mock-treated transgenic plants and biotin-treated nontransgenic (NT) WT plants were used as controls. Axes present log values of the peptide intensity ratio between indicated samples. Significantly enriched protein candidates were selected using cutoffs PSM > 1, *P*-value < 0.05 (liner model F-tests), and fold-change > 4 compared to both controls and are labeled by red dots. Protein candidates from outer mitochondrial membrane (OMM), intermembrane space (IMS), and inner mitochondrial membrane (IMM) are outlined and labeled by blue, red, and green text, respectively. (*B*) Heatmap of normalized average PSM values of OMM, IMS, and IMM proteins identified by PMF1 with high confidence. Colored dots in the heatmap indicate statistical significance (red for *P*-value ≤ 0.01 and orange for *P*-value ≤ 0.05, linear model F-tests) compared to both controls. Enrichment of OMM, IMS, and IMM proteins in PMF1 proxiome using Gene Ontology (GO) enrichment analysis is shown below. (*C*) Schematic diagram of PMF1 localization in the OMM, its topology, and proximal proteins identified. (*D*) *N. benthamiana* leaf epidermal cells coexpressing mCherry-TOM20 with combinations of BiFC constructs, including nYFP-PMF1, PMF1-nYFP, MTS-cYFP, and free cYFP. (Scale bar, 5 μm.) (*E*) Mitochondria isolated from *N. benthamiana* leaves expressing PMF1-HA-TurboID or HA-TurboID-PMF1 were divided into three equal aliquots and treated with 0, 2, or 4 µg/mL Proteinase K for 30 min. Western blots were probed with anti-HA antibodies. Band intensities were quantified and normalized to the mock treatment.

To determine the proxiome and topology of PMF1 on the OMM, we performed proximity labeling proteomics using PMF1 as bait. Proximity labeling was conducted using 1-wk-old *35S::PMF1-BioID2* transgenic Arabidopsis seedlings. Biotinylated proteins were affinity-purified using streptavidin beads, subjected to on-bead trypsin digestion, and analyzed by label-free quantitative mass spectrometry (LFQMS). Three biological replicates were performed, with mock treated and biotin-treated nontransgenic plants serving as controls. Applying thresholds of fold-change > 4 and *P*-value < 0.05 relative to both controls, we identified 118 PMF1-specific preys (Fig. 6*A* and Dataset S1).

Consistent with its mitochondrial localization, PMF1 proximal proteins are highly enriched in known mitochondrial proteins, including components of the mitochondrial protein translocation machinery (TIM/TOM), sorting and assembly machinery complex (SAM), mitochondrial intermembrane space assembly (MIA) proteins, voltage-dependent anion channel (VDAC), and respiratory chain complexes I–V (Fig. 6 *B* and *C*). These proteins are widely distributed across the OMM, IMM, and intermembrane space (IMS). Given that PMF1 is an OMM protein with a single transmembrane domain and that the BioID2 tag was fused to PMF1’s C-terminus, we infer that PMF1’s N terminus is cytoplasm-oriented while its conserved C-terminus faces the IMS (Fig. 6*C*).

To confirm PMF1’s topology on the OMM, we transiently coexpressed either PMF1-nYFP or nYFP-PMF1 with MTS-cYFP or free cYFP, which localize to the mitochondrial interior or cytosol, respectively (Fig. 6*D*). mCherry-TOM20 was coexpressed as a mitochondrial marker. Strong complemented YFP fluorescence was detected in cells coexpressing PMF1-nYFP with MTS-cYFP and in those coexpressing nYFP-PMF1 with free cYFP (Fig. 6*D*), suggesting that PMF1 resides on the OMM with its N terminus in the cytosol and its C-terminus in the IMS.

Last, we transiently expressed *35S::PMF1-HA-TurboID* or *35S::HA-TurboID-PMF1* in *N. benthamiana*. Mitochondria were isolated from transformed tissues and divided into three equal aliquots for Proteinase K digestion. The N terminus of PMF1 was readily degraded, whereas the C-terminus was protected from digestion (Fig. 6*E*), indicating that the C-terminus is shielded by the OMM and further supporting the proposed PMF1 topology. The basal degradation observed in both samples may result from the presence of damaged mitochondria during sample preparation.

Together, these data suggest that PMF1’s N terminus faces the cytoplasm and potentially mediates mitochondrial contact and fusion.

### PMF1’s N Terminus Exhibits Oligomerization and Phase Separation Necessary for Megamitochondria Formation.

**Fig. 7.**
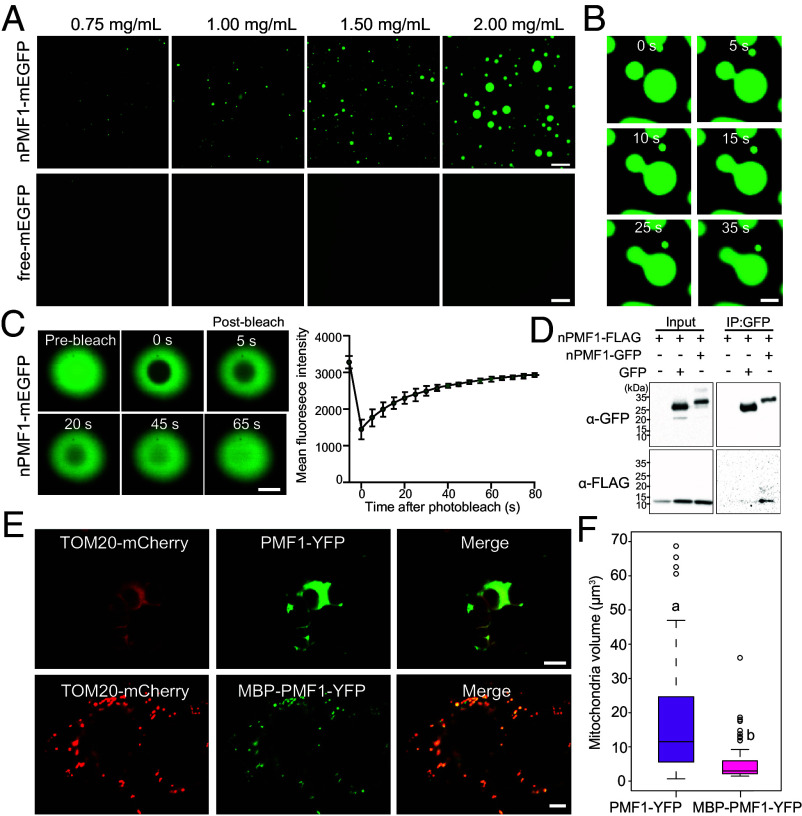
PMF1 undergoes phase separation through its N terminus. (*A*) Formation of in vitro droplets by purified nPMF1-mEGFP protein at indicated concentrations. (Scale bars, 5 μm.) (*B*) Time-lapse microscopy showing representative fusion events of two nPMF1-mEGFP droplets in vitro. Time 0 indicates the start of recording. (Scale bar, 2 μm.) (*C*) FRAP analysis of in vitro nPMF1-EGFP droplets. Time 0 indicates the start of photobleaching. The FRAP recovery curve is shown on the *Right* (n = 10 droplets). (Scale bar, 10 μm.) (*D*) nPMF1-FLAG was transiently coexpressed with free GFP or nPMF1-GFP in *N. benthamiana.* Total protein was extracted, and immunoprecipitation was performed using GFP-Trap beads followed by immunoblotting with anti-FLAG and anti-GFP antibodies. (*E*) TOM20-mCherry was transiently coexpressed with PMF1-GFP or MBP-PMF1-GFP in *N. benthamiana*. (Scale bar, 5 μm.) (*F*) Mitochondrial volume in *N. benthamiana* plants coexpressing *35S::mCherry-TOM20* with *35S::PMF1-YFP* or *35S::MBP-PMF1-YFP*. Statistical significance was determined using a heteroscedastic, two-tailed *t* test and a *P*-value threshold of 0.05. n > 100 for all samples.

The cytoplasmic N terminus of PMF1 contains an IDR (Fig. 1 *D* and *E*). Given that IDRs are established regulators of liquid–liquid phase separation (LLPS) (55), that we frequently observed PMF1 aggregation at megamitochondria boundaries (Fig. 3), and that PMF1 was previously identified in proteome-wide analyses as forming large membrane-associated complexes (56), we further investigated whether the PMF1 N terminus (nPMF1) undergoes LLPS. In vitro LLPS assays using purified nPMF1-mEGFP expressed in insect cells (*SI Appendix,* Fig. S6*A*) revealed a strong propensity for phase separation, with spherical droplets forming at increasing protein concentrations (Fig. 7*A*). These droplets showed high sensitivity to 1,6-hexanediol, an aliphatic alcohol that disrupts LLPS (*SI Appendix,* Fig. S6 *B*–*D*). Time-lapse imaging revealed the dynamic nature of condensates formed by nPMF1-mEGFP, demonstrating their ability to fuse through necking and relax upon contact (Fig. 7*B*). Fluorescence recovery after photobleaching (FRAP) analysis of nPMF1 droplets revealed rapid signal recovery within 80 s, characteristic of dynamic molecular exchange commonly observed in biomolecular condensates undergoing LLPS (Fig. 7*C*). Furthermore, we showed that nPMF1 undergoes intermolecular interactions using coimmunoprecipitation assays (Fig. 7*D*), suggesting that PMF1 LLPS is driven by protein–protein interactions. These findings collectively demonstrate PMF1’s capacity for LLPS through its N-terminal IDR.

To assess the functional importance of nPMF1 LLPS in vivo, we fused Maltose Binding Protein (MBP), a solubility-enhancing tag, to PMF1’s N terminus to disrupt LLPS of this domain. Remarkably, when expressed in *N. benthamiana* leaf epidermal cells, MBP-PMF1-YFP almost completely abolished the mitochondrial enlargement induced by PMF1-YFP (Fig. 7 *E* and *F*), strongly supporting that nPMF1 oligomerization and LLPS is necessary for PMF1’s mitochondrial fusion-promotion activity. Collectively, these data support a model wherein PMF1 proteins interact through their cytosol-facing N termini to promote LLPS at the intermitochondrial contact site, potentially enhancing membrane proximity, recruiting fusion factors, or inducing membrane curvature to enable fusion.

### The PMF1 N Terminus Is Necessary and Sufficient to Promote Mitochondrial Fusion.

**Fig. 8.**
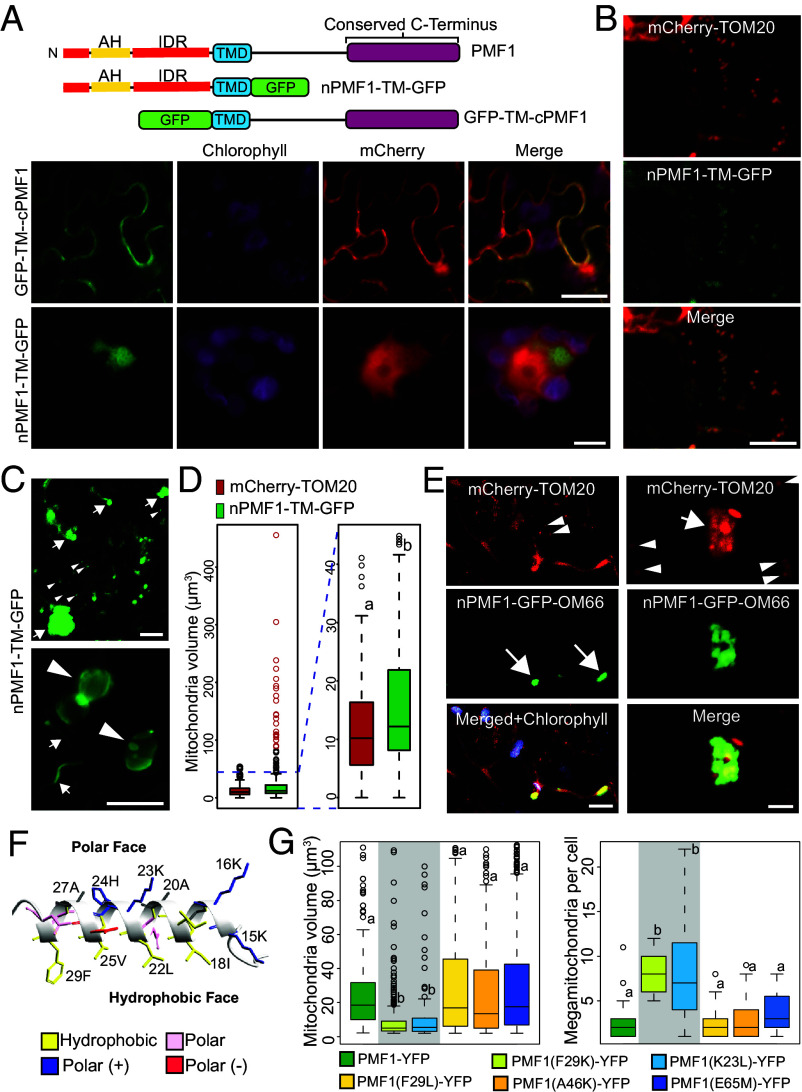
PMF1’s N terminus is sufficient to promote mitochondrial fusion in planta. (*A*) Schematic diagram of PMF1 truncations and their localization when transiently expressed in *N. benthamiana.* Free mCherry was coexpressed as a nuclear marker. [Scale bars, 20 μm (*Upper*) and 5 μm (*Lower*).] (*B*) Colocalization of nPMF1-TM-GFP and OMM marker mCherry-TOM20 when transiently coexpressed in *N. benthamiana*. (*C*) Transient expression of nPMF1-TM-GFP in *N. benthamiana*. Normal and abnormal mitochondrial structures were labeled with arrowheads and arrows, respectively, in the *Upper* panel, (Scale bar, 20 μm) Sac-like and tube-like structures were labeled with arrowheads and arrows, respectively, in the *Lower* panel. (Scale bar, 5 μm.) (*D*) Mitochondrial volume in *N. benthamiana* plants expressing mCherry-TOM20 or nPMF1-TM-GFP. Megamitochondria with volumes over 80 μm^3^ are colored in red. Statistical significance was determined using a Student’s *t* test and a *P*-value threshold of 0.05. n > 200 for all samples. (*E*) Formation of abnormally large mitochondrial structures when nPMF1 was fused to the cytoplasmic end of OMM protein OM66. nPMF1-GFP-OMM66 was coexpressed with mCherry-TOM20 in *N. benthamiana*. Normal and abnormal mitochondrial structures were labeled with arrowheads and arrows, respectively. [Scale bars, 10 μm (*Left*) and 5 μm (*Right*).] (*F*) Schematic of PMF1’s N-terminal amphipathic helix predicted by AlphaFold. (*G*) Mitochondrial size (*Left*) and the number of megamitochondria per cell (*Right*) were quantified in *N. benthamiana* cells transiently expressing the indicated PMF1-YFP point-mutation variants. Mitochondrial volume statistical significance was determined using a Student’s *t* test and a *P*-value threshold of 0.0001. n > 100 for all samples. Megamitochondria count per cell statistical significance was determined using a Mann–Whitney *U* test and a *P*-value threshold of 0.05. n > 16 for all samples.

To further elucidate the roles of PMF1’s N terminus in mediating mitochondrial fusion, we created GFP fusions of PMF1 truncations, consisting of the TMD with either the N- or C-terminus (Fig. 8 *A*, *Upper* panel). GFP-TM-cPMF1 exhibited cytoplasmic localization, indicating loss of mitochondrial targeting (Fig. 8 *A*, *Lower* panel). In contrast, nPMF1-TM-GFP still induced formation of large aggregates (Fig. 8 *A*, *Lower* panel) and colocalized with mitochondria labeled by mCherry-TOM20 (Fig. 8*B*). However, these structures morphologically differed from the full-length PMF1-induced megamitochondria in that they were more amorphous and that the size of individual membrane compartments, when discernable, were typically smaller (Fig. 8 *C*, *Upper* panel). We also observed multiple tube-like structures (Fig. 8 *C*, *Lower* panel), resembling artificial mitochondrial networks resulting from overexpression of fusion-related genes or knockouts of fission-related genes in yeasts (29) and in plants (44, 47, 54, 57). The average mitochondrial volume in *N. benthamiana* cells transiently expressing nPMF1-TM-GFP was 23.81 μm^3^, representing a 96.3% increase compared to cells expressing the OMM marker mCherry-TOM20 (Fig. 8*D*). These findings suggest that nPMF1-TM is sufficient to promote mitochondrial fusion in vivo, which aligns with the PMF1 topology we elucidated.

To substantiate the role of PMF1’s N terminus in promoting mitochondrial fusion, we created a chimeric protein by fusing nPMF1-GFP to the cytoplasmic terminus of OM66, an integral OMM protein (*SI Appendix,* Fig. S7*A*) (58, 59). While GFP-OM66 exhibited typical mitochondrial localization and morphology when transiently expressed in *N. benthamiana* (*SI Appendix,* Fig. S7*B*), the nPMF1-GFP-OM66 chimera was sufficient to trigger extensive mitochondrial aggregation and enlargement (Fig. 8*E* and *SI Appendix,* Fig. S7*C*), nearly doubling the median mitochondrial size. This demonstrates that the PMF1 N-terminal domain alone can promote fusion events when targeted to mitochondria in plant cells. These observations offer compelling evidence that the PMF1 N terminus possesses the capacity to promote mitochondrial membrane fusion in plant cells.

In addition to its multiple disordered regions, the PMF1 N terminus also contains a predicted amphipathic helix (Fig. 1*D* and *SI Appendix,* Fig. S1*D*). Amphipathic helices facilitate membrane fusion in multiple endomembrane systems (32, 60, 61). Often, this is achieved by inserting their hydrophobic residues into the outer leaflet of the phospholipid bilayer, disrupting the tight packing of lipid heads and leading to membrane curvature and destabilization, which reduces the activation energy of membrane fusion (62, 63). Notably, human mitofusins MFN1/2 also contain lipid-bilayer-binding amphipathic helices, and disruption of these helices greatly diminishes MFN’s ability to promote mitochondrial fusion (32–34).

To determine whether PMF1’s amphipathic helix is also necessary for promoting mitochondrial fusion, we introduced F29K and K23L point mutations into PMF1-YFP, which disrupt helix amphipathicity (Fig. 8*F*). Expression of PMF1(F29K)-YFP and PMF1(K23L)-YFP in *N. benthamiana* leaf epidermal cells resulted in a significant reduction of median mitochondrial volume compared to PMF1-YFP (Fig. 8*G*). Meanwhile, both mutants produced a greater number of smaller megamitochondria per cell relative to PMF1-YFP–expressing cells (Fig. 8*G*). Because total mitochondrial volume is unlikely to change based on our previous observations, the presence of more but smaller megamitochondria indicates impaired fusion activity.

To verify that the observed disruptions to PMF1’s fusion-promoting capacity were specifically due to perturbation of the amphipathic helix, we created point mutants F29L, a conservative mutation that does not reduce helix amphipathicity, as well as A46K and E65M, radical mutations outside the amphipathic helix. Cells expressing PMF1(F29L)-YFP, PMF1(A46K)-YFP, or PMF1(E65M)-YFP were not significantly different from those expressing PMF1-YFP for either mitochondrial volume or megamitochondria count per cell (Fig. 8*G*). These data highlight the importance of PMF1’s N terminus amphipathic helix in promoting mitochondrial membrane fusion in plant cells.

## Discussion

**Fig. 9.**
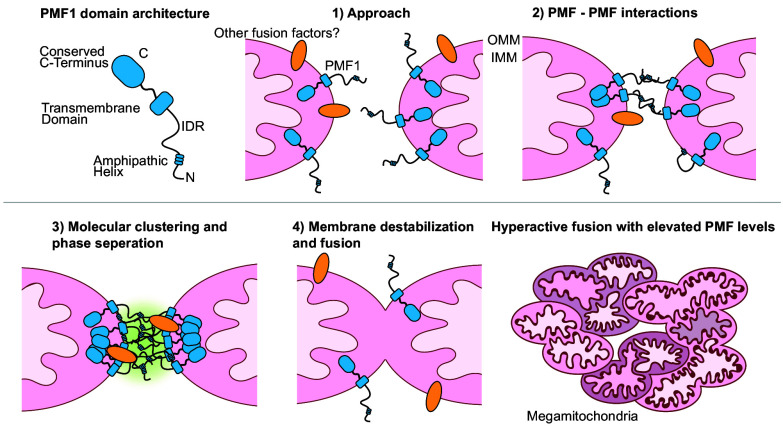
Model for the role of PMF1 in promoting mitochondrial fusion. (1) The N-terminal domain of the PMF1 protein orients toward the cytoplasm at the OMM. (2) This N-terminal region mediates intermolecular interactions, facilitating tethering between approaching mitochondria. (3) PMF1 protein concentrates at intermitochondrial contact sites, triggering local phase separation. Phase separation and amphipathic helix–membrane interactions potentially modify membrane properties to promote fusion. Other fusion factors may be recruited to the intermitochondrial contact site. (4) Outer membrane fusion completes.

While mechanisms of mitochondrial fission and fusion have been characterized in fungi and metazoans with increasing detail over the past several decades, regulatory mechanisms of plant mitochondrial fusion have largely remained a mystery. Our findings reveal an intriguing factor promoting plant mitochondrial fusion while highlighting important questions for future investigation. We propose a model wherein PMF proteins, anchored in the OMM with a cytoplasm-facing N terminus, mediate intermitochondrial contact through intermolecular interactions. The subsequent enrichment of PMF proteins at mitochondrial contact sites promotes molecular condensation and phase separation. Both phase separation and the PMF1 amphipathic helix potentially modify local membrane stability and curvature to promote fusion (Fig. 9). Additional fusion-promoting proteins, including Miro1 and Miro2, are likely involved in this process, and the mechanistic relationship between these fusion-related factors should be the subject of future research.

Plant mitochondria typically appear as discrete puncta whereas animal mitochondria display elongated, tubular, or networked mitochondrial morphologies. This disparity reflects a unique equilibrium between fusion and fission processes in plant cells, in which fission is dominant over fusion. Such distinctions may explain the lack of an overt phenotype and relatively subtle alterations in mitochondrial morphology observed in *pmf1 pmf2* double mutants under standard conditions. Alternatively, functional redundancy cannot be excluded, and unidentified proteins may compensate for the loss of PMF proteins in plants. It is also possible that PMFs act as promoters of mitochondrial fusion without being strictly required, such that fusion can still occur in their absence, potentially mediated by additional fusion factors. Notably, knockout lines of plant mitofusin candidate Miro2 likewise show no phenotype under these conditions (52).

The functional importance of PMF proteins in mitochondrial fusion becomes evident under hypoxic stress, when fusion activity is elevated. Impaired fusion in response to hypoxia in the *pmf1 pmf2* mutant, as well as their reduced survival after hypoxia stress, reinforces that mitochondrial dynamics are a critical component of the cellular metabolic stress response. Preliminary evidence indicates that *miro2* knockouts may also have increased sensitivity to hypoxia, but additional research is needed to more thoroughly explore this phenotype (64). Moreover, the enhanced long-heat stress tolerance of the *pmf1 pmf2* mutant underscores the importance of mitochondrial dynamics in the plant heat stress response. Interestingly, PMF1 overexpression lines do not show the high sensitivity to long-heat stress seen in fission defective mutants, indicating that mitochondrial size alone is not responsible for increased heat sensitivity. The molecular mechanism through which mitochondrial morphology modulates long-heat stress tolerance remains unknown and warrants further investigation.

The identification of PMF1 by proximity labeling using a nuclear envelope protein highlights the underexplored relationship between the nuclear and mitochondrial membrane. While multiple studies have documented the enrichment of mitochondrial aggregates in the perinuclear region of multiple model organisms (14, 65, 66), its functional relevance remains unclear. Given that nuclear envelope proteins facilitate mitochondria–ER contacts known to regulate mitochondrial dynamics, further investigation of nuclear envelope–mitochondria interactions may reveal additional regulatory mechanisms governing mitochondrial fusion and fission events (16, 67, 68).

Our current data indicate that overexpression of PMF proteins is sufficient to promote mitochondrial fusion in vivo; however, this does not exclude the possibility that additional factors, such as a GTPase, are required for membrane fusion. Recent studies have demonstrated membrane-associated phase-separation on multiple endomembrane surfaces, including the Golgi (69), endoplasmic reticulum (70), lysosomes, (70) peroxisomes (70), and at membrane-membrane or membrane-liposome junctions (71, 72). Notably, higher-order oligomerization or pH-induced protein disordering is critical for fusion-induction activity of numerous viral proteins (73, 74). Membrane-associated IDRs and their phase-separation, similar to that observed in the N terminus of PMF1, have been shown to drive membrane curvature (75, 76), which destabilizes membranes, often as a precursor to membrane fusion. However, mechanistic details of how membrane associated LLPS may influence membrane fusion require further study. It is possible that PMF1/2 operate in concert with GTPases linked to plant mitochondrial fusion, such as Miro1/2. Further investigation is necessary to elucidate the precise interplay between these fusion factors and how they may work together to complete the mitochondria fusion process in plants.

While our study demonstrates the potent mitochondrial membrane remodeling capabilities of PMFs, the distinct functions of PMF1 and PMF2 in mitochondrial fusion remain to be fully elucidated. The presence of two or more PMF homologs in most land plant species suggests functional divergence. This is further supported by the observation that overexpression of PMF1 and PMF2 results in distinct membrane architectures in Arabidopsis. It is plausible that PMF1 and PMF2’s unstructured N termini can also undergo dynamic homo- and heterodimerization, similar to the interplay between MFN1 and MFN2 in animal systems, to regulate mitochondrial fusion dynamics in plants. Further, the importance of the PMF1 N terminus amphipathic helix in its fusion-induction capability is reminiscent of the critical MFN1/2 cytoplasmic amphipathic helices. However, the divergence of mitochondrial fusion mechanisms between plants and animals limits our ability to make meaningful inferences between model systems.

A notable aspect of PMF proteins and their homologs is the presence of a highly conserved C-terminal domain. While our findings demonstrate that this region is not essential for OMM localization or fusion promotion, its widespread conservation across land plants raises intriguing questions about its functional significance. The morphological difference between megamitochondria induced by nPMF1-GFP and PMF1-GFP indicates that the PMF1 C-terminus indeed plays a role in remodeling mitochondrial membrane morphology, but the precise activity of this IMS-oriented domain remains to be elucidated.

Lastly, the absence of PMF homologs in algae suggests a potentially divergent mechanism for mitochondrial fusion in these organisms. This disparity underscores the diverse strategies evolved for mitochondrial fusion even within the plant kingdom. Future investigations into these aspects will undoubtedly provide valuable insights into the convergent evolution and functional diversification of mitochondrial fusion mechanisms in eukaryotes.

## Methods

### Phylogenetic Analysis.

To construct the phylogenetic tree, the PMF1 protein sequence from TAIR 10 was used as the query for BLAST search (e-value < 10^-4^). Homologous sequences were retrieved from 14 eukaryotic species and are listed in Dataset S2.

### Molecular Cloning.

All cloning was performed with Vazyme’s InFusion technology (ClonExpress II One Step Cloning Kit, Vazyme). Inserts were PCR amplified from cDNA with PrimeSTAR GXL polymerase (Takara Bio Inc.) and then extracted from a 1% agarose gel with the EZ-10 Spin DNA Gel Extraction Kit (Bio Basic) and inserted into a plasmid backbone cut with restriction enzymes. The native promoter of *PMF1* and *PMF2* (1,500 bp upstream of the start codon) was amplified and cloned into empty vector pEG100. Point mutations were generated using a QuikChange Site-Directed Mutagenesis Kit (Agilent Technologies). The full list of PCR primers is provided in Dataset S3.

### Mitochondrial Volume Analysis and Counting.

Z-stacks were compiled from images with a step size of ½ Nyquist resolution and analyzed in Imaris Version 10.1. Mitochondrial volume was calculated from surfaces mapped with background subtraction and a morphological split. Surfaces were manually curated to remove those that did not align with visual assessment of the fluorescence signal. Mitochondrial counting was performed manually, and total mitochondrial volume was calculated by multiplying average volume and count, with propagation of error used for error bars and statistics.

### Mitochondria Fusion Rate Assay Using Photoconvertible mEOS.

pQE32-pr-mEosFP (mEosFP-V69T) was a gift from Periklis Pantazis (Addgene plasmid # 99213). Leaves transiently expressing MTS-mEOS were sprayed with dexamethasone 24 h after transformation and analyzed after an additional 16 h. Dex-treated cells were screened for CFP fluorescence to verify PMF1 expression. Photoconversion was performed using a 400 nm laser. Z-stacks were compiled immediately after photoconversion and 10 min after photoconversion. Fusion rate was calculated by subtracting the number of mitochondria displaying both green and red fluorescence immediately after photoconversion from the number observed 10 min later, and then dividing this difference by the total number of photoconverted mitochondria. This result was then divided by ten to yield the number of fusion events per mitochondria per minute.

### Hypoxia Survival Assay.

Seven-day-old Arabidopsis seedlings were sealed in a BD GasPak EZ airtight container, and oxygen was removed using the GasPak EZ Anaerobe Container System with Indicator (Cat# 260001). Seedlings were removed after 20 h and allowed to recover for three days before imaging and analysis.

### Long Heat Stress Assay and Chlorophyll Quantification.

Ten-day-old Arabidopsis seedlings grown at 22 °C were transferred to a 37 °C growth chamber for 5 d, then allowed to recover for 3 d at 22 °C before analysis. To quantify chlorophyll, seedlings were frozen in liquid nitrogen and homogenized by shaking with metal beads. 1 mL of 100% acetone was added, and samples were incubated at −20 °C for 24 h in the dark. The supernatant OD was measured at 646 nm, 663 nm, and 750 nm.

### In Vitro Protein Phase Separation Analysis.

The nPMF1-mEGFP plasmid in the pET28a backbone was transformed into *E. coli* strain Rosetta (DE3). Transformed cells were cultured at 37 °C until reaching an OD_600_ of 0.6 to 0.8. Protein expression was induced by adding 0.5 mM isopropyl β-D-1-thiogalactopyranoside (IPTG), followed by overnight incubation at 16 °C. Cells were harvested and lysed in lysis buffer (30 mM Tris-HCl, 150 mM NaCl, 20 mM imidazole, and 1 mM PMSF, pH 7.4) using an ultrasonic cell crusher (Scientz-IID). Recombinant protein was extracted by centrifugation at 12,000 g for 1 h at 4 °C and purified using a gravity column containing Ni-NTA Agarose (QIAGEN, Cat# 30210) with a wash buffer (30 mM Tris-HCl, 150 mM NaCl, and 250 mM imidazole, pH 7.4). Protein concentrations were determined using NanoDrop spectrophotometry (IMPLEN NP80). The purified proteins were mixed with LLPS buffer (50 mM Tris-HCl, pH 7.5, and 100 mM NaCl), aliquoted into a 384-well plate, and incubated on ice for 1 h. Droplet dynamics were examined using a Nikon N-SIM S confocal microscope. For FRAP analysis, droplets were bleached using a 488 nm laser at 50% intensity with 100 iterations. Postbleaching images were captured continuously for 3 min at 5 s intervals using NIS-Elements software. Mean fluorescence intensity within the bleached area was analyzed using ImageJ. To assess droplet solubility, recombinant proteins were incubated with 10% 1,6-hexanediol for 30 min at room temperature, followed by turbidity analysis.

### Statistical Analysis.

All statistical analysis was performed in Microsoft Excel Version 18.89.1 or in RStudio Version 2024.04.2 + 764 with R packages ggplot2 and pheatmap. Statistical significance tests were performed using a heteroscedastic, two-tailed *t* test unless otherwise specified. Statistical significance tests for the mitochondrial counts, fusion rate assay, hypoxia survival assay, and chlorophyll quantification was performed using a Mann–Whitney *U* test.

### Other Methods.

Other routine and previously published methods, including plant materials, transient expression in *N. benthamiana*, coimmunoprecipitation and immunoblot analysis, proximity labeling proteomics, confocal microscopy, mitochondrial isolation, and proteinase K protection assay, are described in *SI Appendix*.

## Supplementary Material

Appendix 01 (PDF)

Dataset S01 (XLSX)

Dataset S02 (XLSX)

Dataset S03 (XLSX)

## Data Availability

Raw data files for mass spectrometry analyses have been deposited to the ProteomeXchange Consortium via the PRIDE partner repository (Identifier: PXD039252) (77) and are publicly available. All study data are included in the article and/or supporting information.
