# Supplementary material for: PMF proteins mediate mitochondrial fusion in Arabidopsis
Source: Proc Natl Acad Sci U S A. 2026 Apr 22;123(17):e2601242123. doi: 10.1073/pnas.2601242123 (PMC13123921; doi:10.1073/pnas.2601242123)
Supplement: Supplementary file 1 — Appendix 01 (PDF) [file pnas.2601242123.sapp.pdf]

PNAS

Supporting Information for

PMF Proteins Mediate Mitochondrial Fusion in Arabidopsis

Ryan P. Kenneally, Yu Tang, Wesley J. Bobst, Rui Tong Khor, Lily Garibyan, Neha R. Jag, and Yangnan Gu

Corresponding author: Yangnan Gu
Email: guyangnan@berkeley.edu

This PDF file includes:

- Supporting text
- Figures S1 to S7
- Legends for Datasets S1 to S3
- SI References

Other supporting materials for this manuscript include the following:

- Datasets S1 to S3

Supplemental Information Text

Methods

Transient expression in *N. benthamiana*

Overnight cultures of *Agrobacterium* grown in LB media with appropriate antibiotics were pelleted by centrifugation, resuspended in Induction Media (5 g/L MS salts, 20 g/L sucrose, 10 mM MES, and 200 µM acetosyringone), and incubated at 22°C for 3 hours. The cultures were then adjusted to an OD_{600} of 0.5 and infiltrated into *N. benthamiana* leaves. Leaf tissue was analyzed 48 hours after infiltration or as described.

Co-immunoprecipitation and immunoblot analysis

Total protein was extracted from 0.2 g of flash-frozen *N. benthamiana* leaf tissue, harvested 72 hours post-infiltration, using 400 µL of protein extraction buffer (10 mM Tris-Cl pH 7.5, 150 mM NaCl, 0.5 mM EDTA, 0.5% Nonidet P40 substitute, and protease inhibitor cocktail, pH adjusted to 7.5 at 4°C). The extracts were incubated on ice for 30 minutes and then diluted with an incubation buffer (10 mM Tris-Cl pH 7.5, 150 mM NaCl, 0.5 mM EDTA, pH adjusted to 7.5 at 4°C). The protein mixture was incubated for 1 hour with ChromoTek GFP-Trap agarose beads at room temperature. Beads were washed three times with washing buffer (10 mM Tris-Cl pH 7.5, 150 mM NaCl, 0.05% Nonidet P40 substitute, 0.5 mM EDTA, pH adjusted to 7.5 at 4°C), and bound proteins were eluted by incubation at 95°C for 5 minutes in 2 x SDS loading dye (120 mM Tris-Cl pH 6.8, 20% glycerol, 4% SDS, 0.04% bromophenol blue, and 10% β-mercaptoethanol). Proteins were separated by SDS-PAGE and analyzed by immunoblot using either an anti-GFP antibody (Living Colors, Takara Bio Inc, Cat# 632592, 1:5000) or an anti-FLAG antibody (Monoclonal anti-FLAG antibody, Sigma-Aldrich, Cat# F1804-50UG, 1:1000).

Confocal microscopy

Fluorescence microscopy was performed using a Zeiss LSM880 inverted confocal microscope equipped with a GaAsP detector. For MitoTracker staining, 5-day-old seedlings were incubated at room temperature with 10 nM MitoTracker Red CMXRos (Thermo Fisher) for 10 minutes, then washed three times with distilled water before imaging.For propidium iodide (PI) staining, 5-day-old seedlings were incubated for 10 minutes with 15 µM PI (Invitrogen) at room temperature, followed by three washes with distilled water before imaging. Imaging of seedlings under hypoxic conditions was performed by mounting seedlings in mineral oil between a slide and cover slip and incubating at room temperature in the dark for 2-3 hours.

Proximity labeling proteomics

The BioID2-mediated proximity labeling proteomics method has been previously described.[1,2] Briefly, ten-day-old transgenic seedlings expressing BioID2-tagged PMF1 and wild-type (non-transformant, NT) controls were treated with 50 µM free biotin solution. BioID2-tagged YFP transgenic lines were incubated in water (mock treatment) for 24 hours as another control. Total protein was extracted and subjected to PD-10 desalting column to remove free biotin. The biotinylated protein fraction was incubated with 50 µL of streptavidin-coated magnetic beads (Dynabeads MyOne Streptavidin C1, Invitrogen) at 4˚C overnight, and the beads were washed five times with protein extraction buffer. Bound proteins were eluted by boiling with a loading buffer containing 50 µM biotin and 1% SDS for 30 minutes, separated by SDS-PAGE, and stained with Coomassie blue R-250. Gel bands were digested with trypsin at 37˚C overnight, and peptides

were extracted twice with 1% formic acid in 50% acetonitrile for LC-MS/MS analysis. Gene Ontology enrichment analysis was performed using DAVID Bioinformatics Resources (https://david.ncifcrf.gov/).

Mitochondrial isolation and Proteinase K protection assay

The *35S::PMF1-HA-TurboID* or *35S::HA-TurboID-PMF1* construct was transiently expressed in *N. benthamiana*. Leaf tissue was weighed and suspended in 8 mL/g cold homogenizing buffer (0.4 M mannitol, 1 mM EGTA, 25 mM MOPS–KOH pH 7.8, 10 mM tricine, 8 mM cysteine, 0.1% BSA, and 1% PVP-40). Tissue was homogenized by shaking with metal beads, then centrifuged at 1000 xg for 5 minutes at 4°C to remove debris. The supernatant was moved to a new tube, and centrifugation was repeated until no debris remained. The supernatant was centrifuged at 12000 xg for 20 minutes at 4°C to pellet mitochondria, which were resuspend in 1 mL of wash buffer (0.4 M mannitol, 1 mM EGTA, 10 mM MOPS–KOH pH 7.2). Samples were centrifuged at 1000 xg for 5 minutes at 4°C, and the supernatant was moved to a new tube and centrifuged at 12000 xg for 20 minutes at 4°C. The pellet was resuspended in 450 µl Proteinase K buffer (0.4 M mannitol, 1 mM EGTA, 10 mM MOPS–KOH pH 7.2) and split into three equal aliquots. Proteinase K (Invitrogen, Cat# 25530049) was added to a final concentration of 0, 2, or 4 µg/mL and incubated on ice for 30 minutes. The reaction was stopped by adding PMSF to a final concentration of 2 mM and incubating on ice for 5 minutes. Protein was extracted by adding 5X SDS protein extraction buffer (300 mM Tris-HCl pH 6.8, 50% glycerol, 10% SDS, 0.1% bromophenol blue, and 25% β-mercaptoethanol) and incubating at 95°C for 10 minutes. Proteins were separated by SDS-PAGE and analyzed by immunoblot using an anti-HA antibody (HA-Tag Monoclonal Antibody, GenScript, Cat# 11867423001) and secondary antibody (Goat anti-Mouse IgG (H+L) Secondary Antibody HRP, Thermo Fisher Scientific Cat# 31430).

Figures

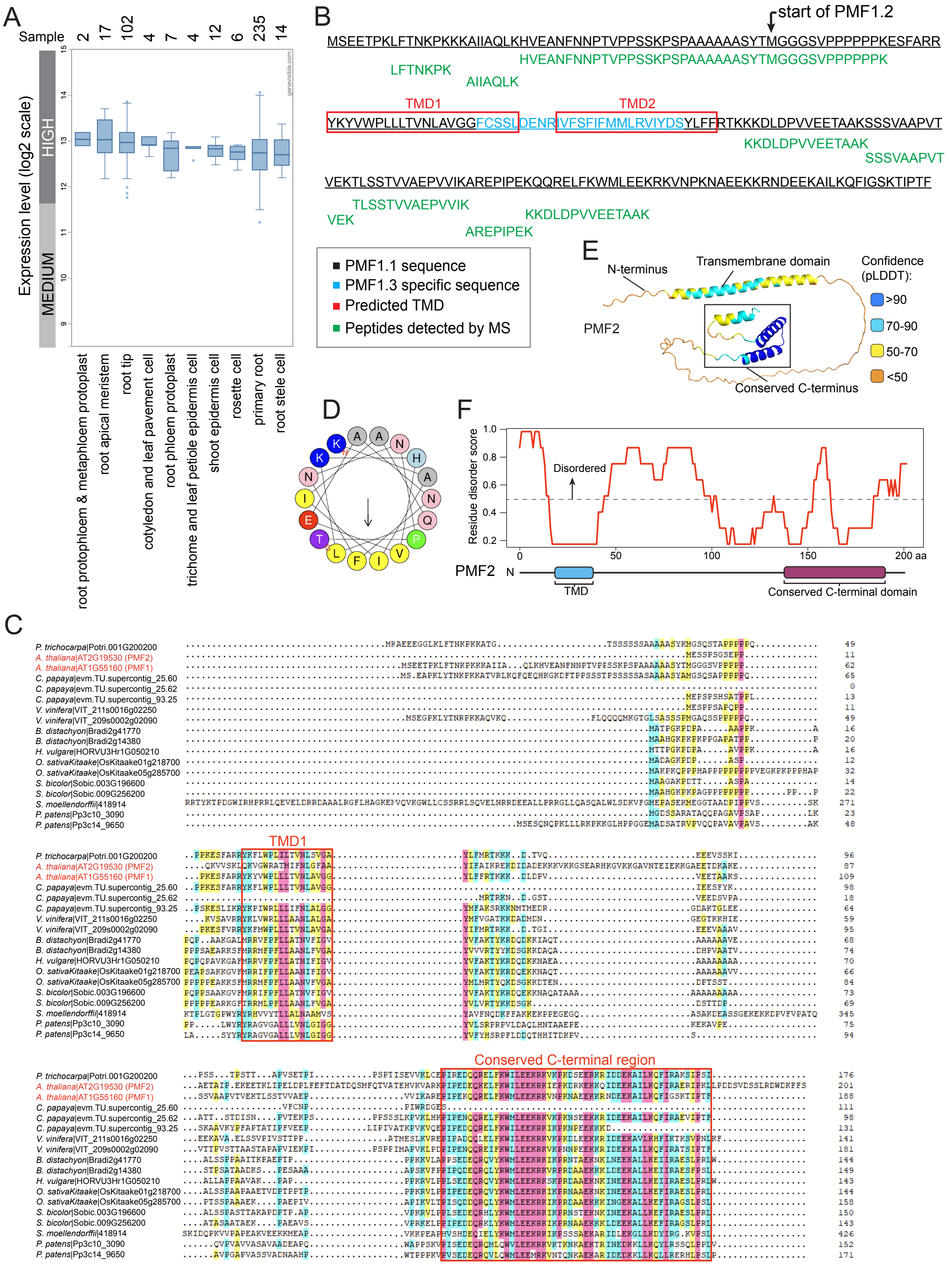

Fig. S1: Characterization of PMF proteins, related to Figure 1.
(A) Expression levels of *PMF1* in Arabidopsis tissues, assessed using GENEVESTIGATOR.
(B) Potential isoforms of PMF1 proteins and endogenous PMF1 peptides detected by mass spectrometry (green text).
(C) Protein sequence alignment of PMF1 and its homologs in a taxonomically diverse subset of land plants.
(D) PMF1 amphipathic helix predicted by Heliquest. Hydrophobicity Score = 0.317 and Hydrophobic Moment Score = 0.434.
(E) Structural model of Arabidopsis PMF2 generated by AlphaFold3, visualized using PyMOL 3.0. Color scheme reflects predicted per-residue confidence scores (pLDDT) from AlphaFold.
(F) Schematic representation of PMF2 protein domain architecture alongside a plot of disorder scores generated by MolPhase. A dashed line marks the disorder threshold at 0.5.

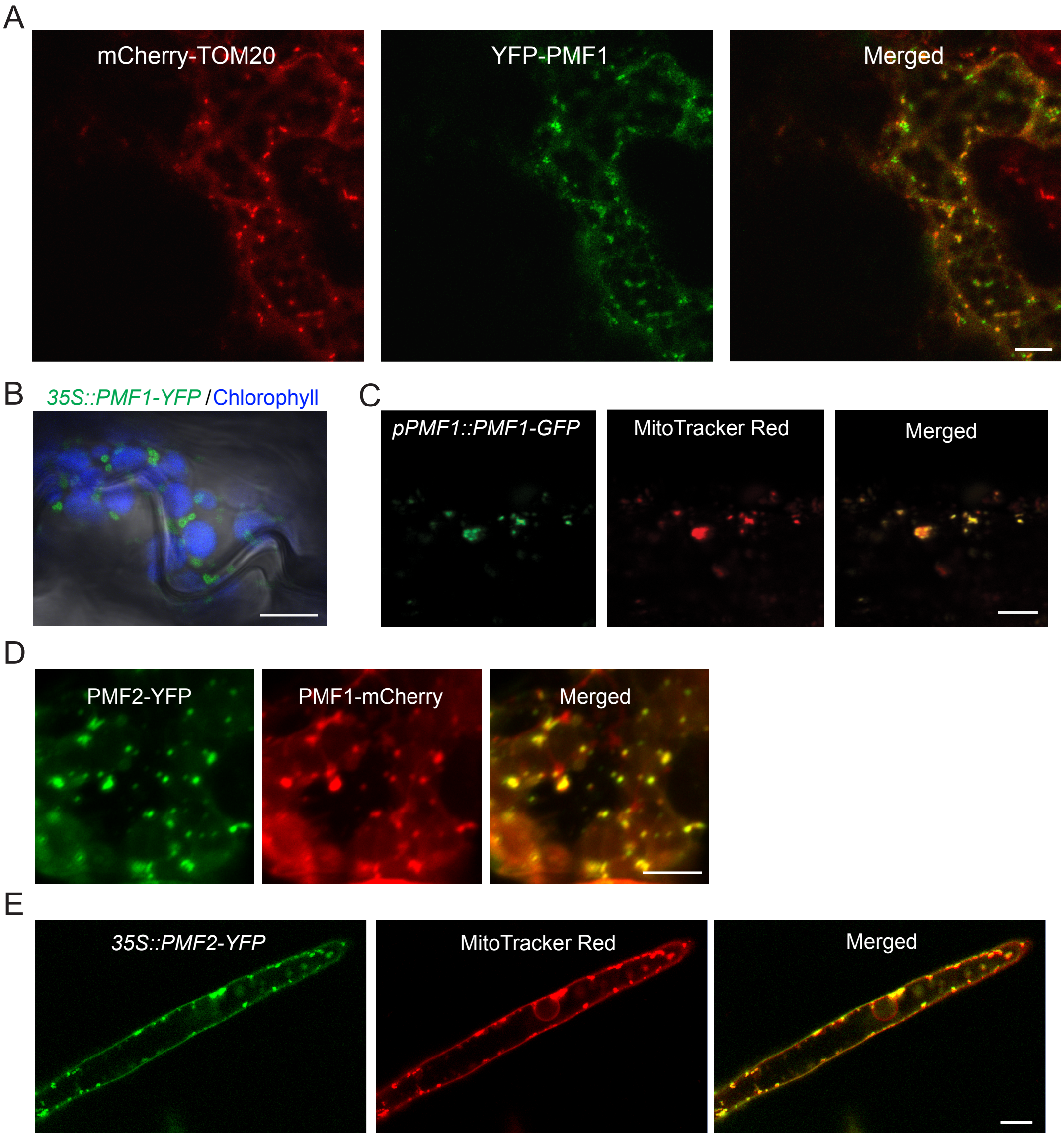

Fig. S2: PMF proteins localize to mitochondria, related to Figure 2.
(A) Transient expression of OMM marker mCherry-TOM20 and YFP-PMF1 in leaves of *N. benthamiana*. Scale bar = 10 μm.
(B) PMF1 localization in *35S::PMF1-YFP* transgenic Arabidopsis seedling. Leaf epidermal cells were imaged. Chlorophyll autofluorescence is psuedo colored in blue. Scale bar = 10 μm.
(C) Hypocotyl epidermal cells of *pPMF1::PMF1-GFP* transgenic Arabidopsis stained with Mitotracker Red CMXRos. Scale bar = 10 μm.
(D) Transient coexpression of PMF2-YFP and PMF1-mCherry in *N. benthamiana*. Scale bar = 25 μm.
(E) Mitochondrial localization of PMF2 in *35S::PMF2-YFP* transgenic Arabidopsis seedlings. Root hairs were imaged. Mitochondria were visualized using MitoTracker Red CMXRos. Scale bar = 10 μm.

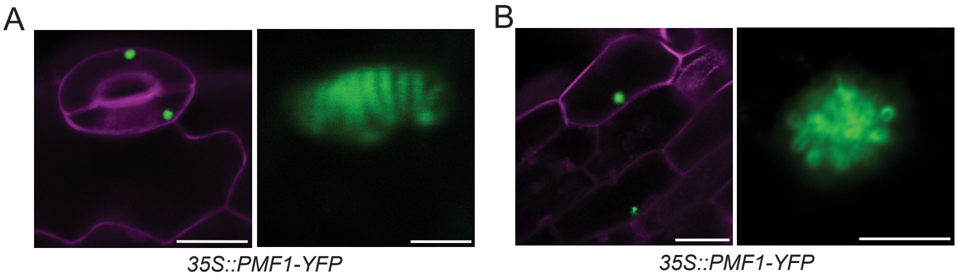

Fig. S3: PMF overexpression leads to the formation of megamitochondria, related to Figure 3.
(A-B) Large mitochondria fusion structures in guard cells (A) and hypocotyl epidermal cells (B) of *35S::PMF1-YFP* transgenic seedlings. Cell walls are stained with PI and psuedo colored in magenta. Higher magnification of an enlarged mitochondrial structure was shown on the right. Scale bars = 10 μm (A, left), 2 μm (A, right), 20 μm (B, left), and 5 μm (B, right).

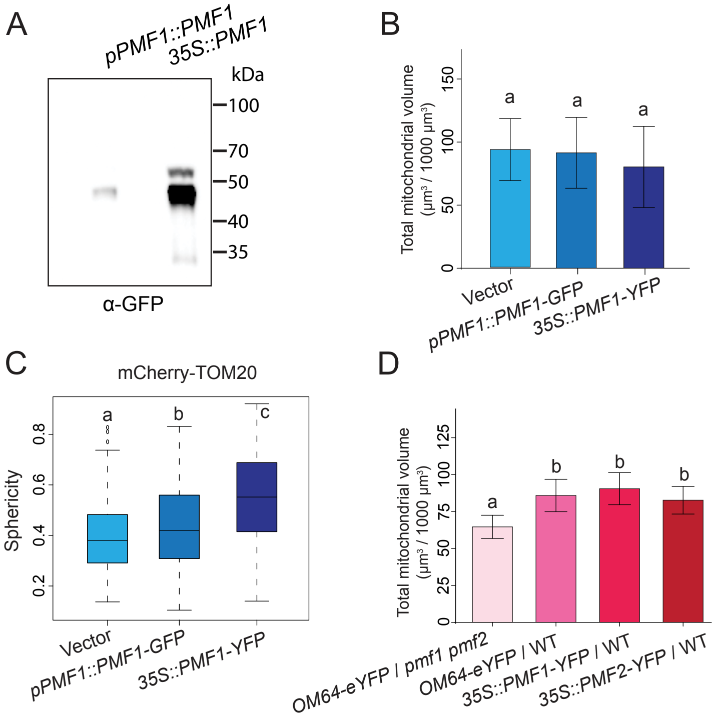

Fig. S4: PMF overexpression promotes mitochondria fusion without changing the total mitochondrial membrane volume, related to Figure 4.

(A) Immunoblot of total protein extracts from equal biomasses of *N. benthamiana* leaves expressing *35S::PMF1-YFP* and *pPMF1::PMF1-GFP* constructs.

(B) Total mitochondrial volume in *N. benthamiana* leaves coexpressing *mCherry-TOM20* with *pPMF1::PMF1-GFP*, *35S::PMF1-YFP* or empty vector. Statistical significance was determined using a Z-test and a p-value threshold of 0.05.

(C) Sphericity of mitochondria in *N. benthamiana* leaves coexpressing *mCherry-TOM20* with *pPMF1::PMF1-GFP*, *35S::PMF1-YFP* or empty vector. Statistical significance was determined using a Student's t-test and a p-value threshold of 0.05. n > 100 for all samples.

(D) Total mitochondrial volumes in *35S::PMF1-YFP, 35S::PMF2-YFP*, and *35S::OM64-eYFP* transgenic Arabidopsis plants. Isogenic *35S::OM64-eYFP* lines in the *pmf1 pmf2* and WT backgrounds were used. Statistical significance was determined using a Z-test and a p-value threshold of 0.05.

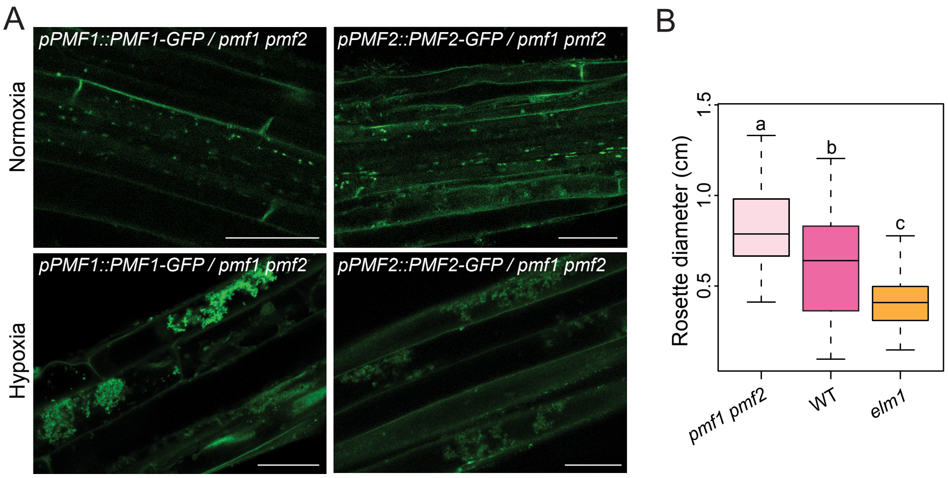

Fig. S5: PMFs impact in hypoxia and long-heat stress tolerance, related to Figure 5.
(A) Root epidermal cells of *pmf1 pmf2* seedlings complimented by *pPMF1::PMF1-GFP* or *pPMF2::PMF2-GFP* under normoxia and hypoxia. Scale bars = 10µm.
(B) Rosette diameter measurements for WT, *pmf1 pmf2*, and *elm1* seedlings subjected to 10 days of standard growth, 5 days of heat treatment at 37°C, and 3 days of recovery. Diameters of plants grown under standard conditions did not differ from each other. Statistical significance was determined using a Student's t-test and a p-value threshold of 0.05. n > 40 for all samples.

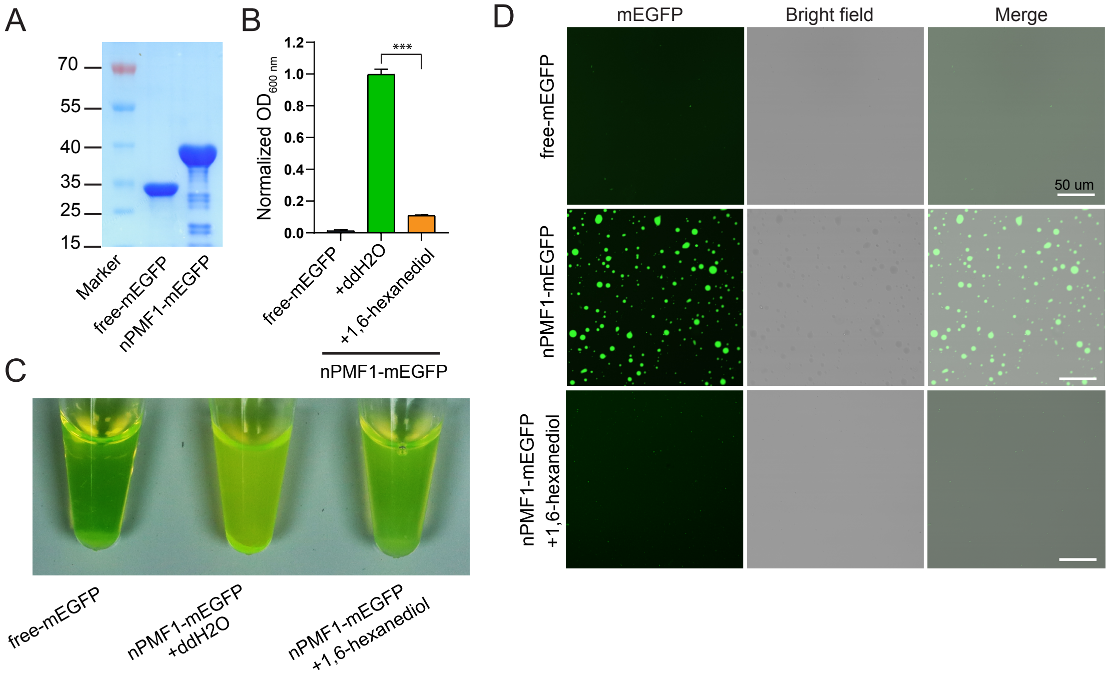

Fig. S6: The N-terminus of PMF1 undergoes liquid-liquid phase separation, related to Figure 7.
(A) Coomassie blue stain of purified free-mEGFP and nPMF1-mEGFP in SDS-PAGE gel.
(B) Normalized OD_{600} of free-mEGFP and nPMF1-mEGFP in solution with or without 1,6-hexanediol. Three technical replicates were used for each sample, and statistical significance was determined using a Student's t-test and a p-value threshold of 0.05.
(C) Purified free-mEGFP and nPMF1-mEGFP in solution with or without 1,6-hexanediol.
(D) Fluorescence microscopy of purified free-mEGFP and nPMF1-mEGFP in solution with or without 1,6-hexanediol.

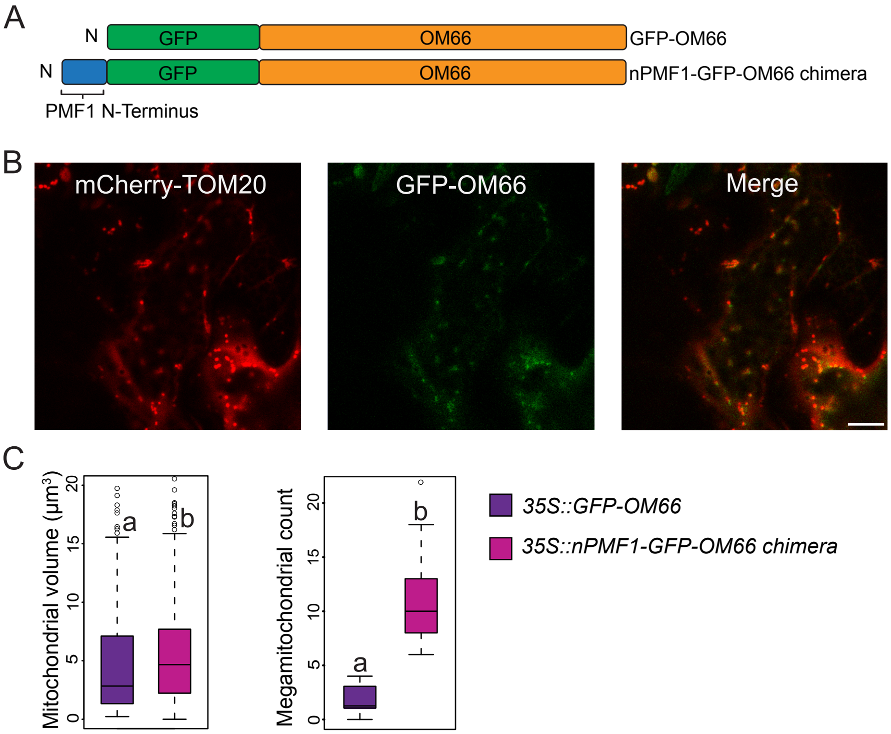

Fig. S7: The N-terminus of PMF1 is sufficient to promote mitochondrial fusion in vivo, related to Figure 8.

(A) Schematic diagram of GFP-OM66 and the nPMF1-GFP-OM66 chimera.

(B) Transient expression of OMM marker mCherry-TOM20 and GFP-OM66 in leaves of *N. benthamiana*. Scale bar = 10 µm.

(C) Mitochondrial volume and the number of megamitochondrial structures per cell when nPMF1-GFP-OM66 was transiently expressed in *N. benthamiana*. Expression of GFP-OM66 was used as control. For mitochondrial volume, statistical significance was determined using a Student's t-test and a p-value threshold of 0.05. $n > 200$ for all samples. For megamitochondrial count, statistical significance was determined using a Mann-Whitney U test and a p-value threshold of 0.05. $n > 18$ for all samples.

Dataset S1: PMF1 proxiome identified by proximity labeling proteomics.

Dataset S2: PMF homolog sequences used for phylogenetic analyses and primers used in this study.

Dataset S3: Primers used in this study.

SI References

1. A. Huang, Y. Tang, X, Shi, M. Jia, J. Zhu, X. Yan, H. Chen, Y. Gu, Proximity labeling proteomics reveals critical regulators for inner nuclear membrane protein degradation in plants. *Nat. Commun.* 11, 3284 (2020)
2. Y. Tang, A. Huang, Y. Gu, Global profiling of plant nuclear membrane proteome in Arabidopsis. *Nat. Plants* 6, 838–847 (2020)
